# Analectic

**Published:** 1836

**Authors:** 


MISCELLANEOUS INTELLIGENCE.

### ANALECTIC, ANALYTICAL, AND ORIGINAL.

### I.—ANALECTIC.

*Ad Sylvas Nuncius.*

1. *A Succinct Account of the Hospitals, Medical Institutions, &c., in Paris.* By JOHN WATSON, M. D., one of the Physicians to the New York Dispensary.—The institutions of Paris for the reception of the sick and infirm, are of two kinds, viz: Hospices, and Hospitals. The Hospices are somewhat similar to our poor houses, but have also infirmaries attached to them, and offer as great a variety of medical and surgical practice, as the hospitals properly so called. These hospices are, in number, eight; the two most important of these are Salpetriere and Bicetre, the first of which is for females only, and contains between five and six thousand inmates; the other is for men, and accommodates about four thousand. Besides the infirm and sick, these two institutions have also an immense number of lunatics, who are classified according to the character of their derangement, and treated with as much attention and kindness as their condition will admit. These institutions are both at some distance from the School of Medicine; the latter, indeed, is two or three miles out of the city, so that they are not attended by the students. They are not, however, useless to the profession; for it was at Salpetriere that M. Cruveilhier collected the materials for his splendid work on Pathological Anatomy. He is, I believe, still pursuing his investigations, and if a sufficiency of materials can conduce to the completion of his work, he can have nothing to complain of.

Of hospitals properly so called, in Paris, there are fourteen, and the average number of patients received in them annually, is between forty-seven and forty-eight thousand. This estimate does not include the military hospitals, nor the Hotel des Invalides, an institution for old disabled soldiers, and of which Baron Larrey is the surgeon-in-chief. The wards of the hospitals are generally very spacious. The principal wards of Hotel Dieu contain each about seventy-five beds. At the Hotel des Invalides, and at La Charite, there are some wards more spacious still. The accommodations for the patients are good; their bedsteads are of iron, their beds are curtained, and kept remarkably clean. The floors of the wards, as of most of the houses in Paris, are paved with tile, which, in the winter season, gives them a cold and cheerless appearance.

*Hotel Dieu* is the oldest, and is considered the principal hospital of Paris; it has accommodations for one thousand patients, of which nearly four-fifths are medical, and the rest surgical cases. It is attended daily by three surgeons and ten physicians, who have each their own wards, and are independent of one another.

*La Charite* is usually considered second in importance, and, like the preceding, it is principally occupied by acute cases; it contains perhaps about five hundred beds, of which the greater part are for medical cases. It is attended daily by two surgeons and four physicians.

*La Pitie*, judging by the preferments of the faculty, is considered third in importance. The medical cases here are usually of a more chronic character. The number of beds is six hundred, of which one hundred and thirty-five are for surgical cases. It is attended by two surgeons and five physicians.

*St. Louis* is principally celebrated for cutaneous and scrofulous affections; it has also an important surgical service, and it so happened that I found a greater variety of fractures and other injuries here, than in any other hospital of Paris. The baths of this hospital are very extensively used, and adapted to every variety of application. About nineteen thousand baths, fumigations and douches, are annually administered to patients within the hospital, and upwards of eighty thousand to out-door patients. The number of beds is about seven hundred, and the patients are under the charge of three surgeons and five physicians.

*The Venereal Hospital* is said to contain about six hundred beds, but of these I only attended the service of M. Ricord, and was led to believe, that, taking into account the greater amount of population in Paris, it has not so much of this disease—at least in hospitals—as may be found in the hospitals in New York.

*The Necker Hospital* is a small establishment, containing only one hundred and twenty-four beds, and resorted to by students only to witness the operations of M. Civiale, who has a service here, and who usually once a week operates, according to his well known process, of breaking up the stone in the bladder.

*The Clinique*, or *Hospital of the Faculty*, is a small and recent establishment, intended principally for the benefit of students about to graduate; here they are examined on the cases before them, and are required to go through all the manipulations that would be necessary, if they had the sole charge of the case themselves. There is here a service of midwifery, of medicine, and of surgery; M. Dubois has charge of the first, M. Rostan of the second, and M. Jules Cloquet of the other.

The preceding are almost the only hospitals that the students resort to for instruction. The Children's Hospital is, however, occasionally attended by some of them. It is intended for the reception of children between two and fifteen years old, but has occasionally some under the age of two years. It contains five hundred and sixty beds, of which sixty-nine are surgical. The situation of this hospital is more pleasant and more retired than that of any of the others; the wards, too, are kept remarkably neat, and every thing is in perfect order. I was somewhat surprised, however, to find children affected with small-pox, measles, and scarlatina, mixed up indiscriminately, and in the same wards with other cases. This I have since found to be the usual practice; and instances of small-pox may at almost any time be found in the other hospitals, and not the least precaution taken to prevent the communication of the disease to the other patients. In short, Paris, with all its accommodations for the sick, has as yet no hospital set apart for contagious diseases.

The attendance of the physicians and surgeons on the hospitals is remarkably systematic; each one—attended by his Interne and Externes, who are required to be present and to answer to their names—pays his visit early in the morning, some commencing at seven, some at eight, but none later than nine. The Interne is usually required to keep a written account of the cases under his care, so as at any moment to be able to give their whole history. The Externes, besides attending to dressings, &c., are required to take down the prescriptions, and afterwards enter them in a tabular form into a book, which the attending physician or surgeon carries through his wards, and which shows him at one view all that had, during his former visits, been ordered to his patients. The notes of the Interne are usually made up from the remarks of the physician or surgeon during the visit; and sometimes he will dictate the appearances and changes in the case, so that not only the Interne, but the class of students in attendance also, may have every means of filling up their notes. Several of the attending physicians and surgeons, after making their morning visit, deliver clinical lectures to the students. These lectures are always drawn from the cases actually under treatment, and afford the lecturer an opportunity, not only of contrasting different cases of the same disease, but also of dwelling more fully on individual affections, and of exhibiting to the students the pathological state of parts, in cases that prove fatal. Nine of these lecturers, viz. four surgical and five medical, are called Clinical Professors, because they are attached to the faculty of the School of Medicine. Their professorship, however, gives them very little consequence in the opinion of their audience; the students never consider whether their instructor be a professor or not, so long as his service is a profitable one, and they are benefitted by his lectures.

Under regulations such as these, it could hardly be otherwise than that the French should become acute observers; and certainly, on those branches to which they have specially directed their attention, they are unequalled. These branches, however, do not embrace the whole, perhaps not even the most important part of the profession, and on many points of it—if I may presume to decide—they are utterly in the dark. They may, nevertheless, have good reason to find fault with us, for striking at their imperfections. They may tell us in reply, that their course of investigation is as yet incomplete; that they are satisfied with perfecting their knowledge on one or two subjects at once; that their ultimate object is the perfection of the profession in all its parts; that they have the means as well as the will to do so; and that the time may not be far distant, when their indications and their treatment shall be even more thoroughly established than their pathology. Such would be their natural comment on those that would complain of them, and certainly it is a reasonable one; for they have begun the investigation of disease at its foundation; they are inquiring of Nature wherein she is affected, and they may one day end by discovering how to set her right. But at present, this last and most essential part of the profession can scarcely be said to be under their consideration; at any rate, they are not advancing it. The generality of them appear to have little confidence in medicine, and what medicaments they use, are, with two or three exceptions, the simplest and least efficient. Amongst the medicinal agents which they appear almost to have condemned, may be enumerated active purgatives, emetics, sudorifics, and alteratives; and those most commonly employed by them are demulcents and diluents; opium and other narcotics are occasionally employed—tonics and stimulants, rarely; but the whole train of mercurial preparations, except in venereal and cutaneous diseases, never. For the purpose of moving the bowels, they scarcely ever administer medicine by the mouth; this object is effected by stimulating enemata. Indeed, they have a peculiar fondness for what they call lavements, and often administer them without any apparent reason; even in diseases of the chest, and in other cases where the bowels are not involved in the disorder—next to the *Bouillon* and *Sirop de Gomme*—the *Lavement Emolient* is the most frequent prescription. With respect to antimonials, I did not find them so freely employed in diseases of the chest, as I had reason to believe; they are nevertheless used occasionally. With respect to venesection, there is great diversity of opinion amongst them; some rarely resorting to it, and others, perhaps, employing it to excess. Their system of regulating the diet of the sick is worthy of imitation; the patient's food is as regularly prescribed as his medicine, and noted down in the same way in the prescription book of the physician.

The surgical practice of Paris, as a whole, is inferior to our own; their bandaging, except in the wards of Baron Larrey, is generally negligent; their mode of putting up fractures, clumsy and imperfect; their operations, in no way better chosen, and never better executed. Taking into account the immense quantity of disease, one is rather surprised to find so small a portion of it arising from accidents. Fractures, dislocations, wounds and other injuries, arising from accidental causes, are comparatively few; and I am pretty well persuaded that there is usually more of such practice in the New York Hospital, than in any two hospitals of Paris. The Parisians are not so subject to accidents, as either the English or Americans; they have not the same amount of intemperance amongst them, they have very few public works in progress, are not exposed to injuries from machinery, and although their streets are narrow, and mostly without side-walks, yet the foot passenger can always run as fast as the fleetest of their cab-horses. But there are some points in their surgery worthy of our attention, and perhaps the most important of these is their mode of studying diseases of the uterus. These they investigate with the strictest care, and in every case where there is any reason to suppose that this organ is affected, they resort to the speculum vaginæ, through which they are enabled both to examine the state of the uterus, and to apply their remedies directly to the part affected.

Among the most popular men attached to the hospitals—beginning, according to French etiquette, with the surgeons—we may enumerate Lisfranc, Velpeau, Roux, Ricord, Civiale, Sanson, J. Cloquet, and the younger Berard; among the physicians, Louis, Chomel, Andral, Bouillaud, Biett, Piorry, Rayer, Dubois, and Rostan. Most of these men already hold conspicuous places in medical literature, and their works are almost as well known in America as in France.

Lisfranc has a service at La Pitie, and is the greatest favorite of the students, his popularity depending as much, perhaps, upon his mannerism, as upon his acknowledged abilities. He endeavors as much as possible to avoid the necessity of operations, and often speaks of having cured by medical treatment, diseases for which others would have operated. He has under treatment a great number of females with diseases of the uterus, and I understood that he is soon to publish a work on these affections. I saw him operate but twice; once for extirpating a small tumor from the parietes of the abdomen, and once for removing the metatarsal bone of the small toe of the left foot. The first of these he had considered a case of cancer, and without any other reason, that I could discover, than that the tumor was attended with acute lancinating pains. The patient was a young man, his disease of several months' standing, and the tumor, when removed, presented no malignant appearances. Just before my arrival, he had operated on a young woman for artificial nose; the integument for this purpose was turned down from the forehead. The patient was still in the hospital, at my departure; but the flap had cicatrized, and the case may be considered fairly successful. I saw but one case of fracture in his wards, and his treatment of this was so characteristic of the man that I am tempted to relate it. The patient, a middle-aged laboring man, had slipped on the ice and broken his leg about midway between the knee and ankle joints. The upper portion of the fractured tibia was very sharp, and when Lisfranc saw it, (the next day after the accident,) the sharp point of the bone had almost penetrated through the integuments. The limb was lying on a pillow, and the contraction of the strong posterior muscles of the leg, had a further tendency to draw the integuments tense over the bone that was pressing upon them from beneath. Raising up the limb, he placed the broken ends in coaptation, but as soon as the leg was at rest on the pillow, the previous displacement again occurred; the limb was again adjusted and placed on the pillow, but with no better result. The same proceeding happened three or four times, and at length Lisfranc, instead of resorting to some sort of apparatus to keep the bones in place, fell into a violent passion with the patient, because he *would* not keep the leg at rest. Every epithet of imprecation, infamy, and reproach, was heaped upon him, but all to no purpose; and although the suffering man literally trembled beneath the scowl of his surgeon, nothing was affected by it—the muscles still continued to contract. Words thus proving useless, he resorted to other measures, and gave the poor fellow a few smart blows over the patella of the broken limb; but these were as unsuccessful as the other means, so that after all his trouble, he had to give up the attempt, and the patient remained as at first until the next day. The displacement was then effectually reduced, and the leg buried beneath a superfluity of thick pads, over which were placed three short straight splints—one on each side, and one in front—and the whole bound tightly together by three or four stout strings. In this position the leg remained for a month, and the splints had not been removed at my last visit.

Velpeau, Professor of Clinical Surgery at La Charite, is attended by a larger class of students than any other surgeon. His service offers a great variety of cases; he is indefatigable in his attention to them, and is, beyond all question, the best clinical instructor in Paris. During the two months of my stay there, he had no capital operations; but I am told that as an operator, he he is not at all distinguished. He is lame of his right hand.

Roux, successor to Dupuytren in the chair of clinical surgery at Hotel Dieu, has also a large class of students attending him. I saw him operate but once, viz. for the cure of hare-lip. In drawing the edges of the wound together, he used the pins. A few days before my first visit, he had filled up a cavity in the cheek of a female patient, by turning a flap of integument over it. The flap was attached to the edges of the wound, in this case also, in the same manner. Roux, as a lecturer, is greatly inferior to the preceding.

Sanson is also at Hotel Dieu; he is at present contending with a number of others for the chair of Clinical Surgery at La Pitie; he has turned his attention, lately, to diseases of the eye, and has been delivering lectures on that subject. I saw him operate for vesico-vaginal fissure, for varicose veins of the leg, and for what was called strangulated hernia. In the first case he applied the actual cautery, defending the adjacent parts by means of the speculum. In the second, the superficial femoral vein was raised up within a fold of the integuments, and pressure made upon it so as to interrupt the passage of blood through it. The pressure was applied by means of two small parallel metallic plates, drawn together by screws. The operation is not a new one; we have the instrument for it in the New York Hospital. After pressure had been applied about six hours, or long enough to produce vesication on one part, the instrument was removed and placed farther down, and so from one place to another along the vein. The result of the case I cannot say. The third case occurred in a female, and was stated to be a strangulated inguinal hernia. A free incision was made over the tumor, along the course of the inguinal canal, quite through the integuments; the second application of the knife passed through the cellular and adipose substance, but as the incision was continued on towards the lower edge of the tumor, it gave vent to a copious gush of purulent matter, in every respect similar to what might be expected from a lumbar abscess. A pint of pus, at least, if not more, was discharged through the opening. M. Sanson found at the bottom of the opening, a sort of ragged, fistulous canal, which he stated to be the intestine, in a state of sphacelus. Of this, however, I was not at all persuaded; the patient had had no hiccough, nor vomiting, so far as I could learn; and I had previously found, in examining a subject that had died of lumbar abscess, a slender canal, almost as regular in its calibre as the small intestine, and extending thus on the surface of the muscles, from the dorsal vertebræ to the groin. After the matter had been all evacuated, M. Sanson examined the appearances of this fistulous canal as carefully as he could, but he was evidently confused, and ordering a poultice to be applied, he left the case without any further light as to its nature. This woman died in the course of a week or so after the operation; and although I made several inquiries, I could not ascertain any thing further concerning her. I found a fractured leg in M. Sanson's wards, carefully secured in splints, and the whole placed on a swing, and elevated ten or twelve inches above the bed.

Ricord, at L'Hospital des Veneriens, has collected many interesting facts on the subject of venereal diseases. He believes that although the true primary syphiltic sore may generally be recognised, it has, nevertheless, no external character that can properly be considered diagnostic. The contagious quality of the virus found in it, he considers the only real diagnostic sign; and even this sign is lost as soon as the ulcerative stage is suspended, and the sore begins to granulate. For the purpose of verifying the disease, he is in the constant habit of inoculating his patients with matter taken from their own sores; he generally applies the matter on the thigh, and sometimes in this way produces disagreeable and obstinate ulcers. He states, however, that he can always interrupt the progress of a chancre, and prevent it from producing constitutional symptoms, when he has an opportunity of seeing it within four or five days of its commencement. To effect this, he cauterizes the sore frequently and freely with argentum nitratum. He may be considered a rational mercurialist, giving calomel and other preparations of mercury in such cases as resist the other means of treatment. He cures chronic urethritis, by the introduction of argentum nitratum once or twice a week, by means of Lallemand's instrument, into the urethra. In the treatment of females, he never neglects to have them examined through the speculum, and most commonly finds chancres or abrasions about the os tincæ and neck of the uterus. He makes a thorough examination of all the females, before his class, once a week.

Civiale has a small ward in the Necker Hospital, and operates every Saturday on such of his patients as are in a condition to undergo the process. During my stay, I attended on his operations five or six times. He had in all this period but three patients, two of whom were discharged cured, and the third, although still in the hospital, had been relieved of almost every part of his calculus. The first case, a robust man about fifty years old, was cured, after undergoing the operation three times. The first trial had excited some irritability of the urethra and swelling of the testicle; but after these were subdued, the patient submitted to the operation without any apparent pain, and to all appearance was effectually cured. In the second case, the urethra was rather contracted, and the bladder irritable. The patient was a delicate old man, perhaps about sixty-five; he bore the operation without much expression of pain, and was cured in five trials. The third case was a man of lymphatic temperament, about the same age as the preceding. He had undergone the operation eleven times, and every time with fear and trembling. The stone, in this case, was excessively hard; its fragments could not be indented with the finger nail. He had preserved all the fragments voided; they might have weighed two ounces, or perhaps three, and some of them were twice as large as peas. M. Civiale sometimes uses his own, and sometimes Hourteloup's instrument. The latter, however, is seriously objectionable. It is apt to get clogged with the debris of the stone; the blades are thus prevented from coming close together, and on reaching the meatus externus, they are arrested by the narrowness of the canal, and it is necessary to cut the meatus, in order to remove the extremity of the instrument. Since my return to London, I have seen an improvement on this instrument, by which this objection to it may be obviated.

Among the physicians enumerated above, Louis and Andral are at La Pitie. The latter I heard lecture a few times at the School of Medicine, where he draws an overwhelming class of students; but I had not an opportunity of meeting him at the hospital. During the winter season, his attendance there is not regular. Louis' manner of searching out his patients' affections, is worthy of admiration; a good description of his method may be found in the Life of Dr. Jackson, by his father. His service is mostly made up of chronic cases, and he is followed by a great number of students, who are endeavoring to become familiar with the stethoscopic signs of disease.

Chomel is Professor of Clinical Medicine at Hotel Dieu. He investigates the affections of his patients on much the same plan as Louis, but never uses the stethoscope; instead of this, he always applies his ear immediately to the chest. His cases are generally acute, and his service offers a great variety.

Bouillaud, at La Charite, makes a greater display of accuracy in examining his patients, than either of the preceding; he not only carries his stethoscope, but also his test-paper, for examining the urine, and his tape, for measuring the relative size of different parts of the body and limbs. He has recently advanced some new opinions on the subject of rheumatism, an account of which he has already published. He depletes more freely than any other physician of Paris, and there have recently been some warm discussions between him and Louis, on the propriety of blood-letting.

In the French system of professional preferment, every advancement, from the dressership upwards, is only to be acquired by *concours*, public examination and public competition. This method speaks for itself; it is the only true way to secure to industry and merit their proper recompense.

A few remarks might perhaps not be amiss, on the advantages the American student may expect to obtain, and the objects he should attend to, in visiting Paris. The greater number of American physicians who resort to the French hospitals, leave home immediately after receiving their degrees, and very frequently without having attended to the hospital practice in their own country. Under these circumstances, they are ignorant of what is excellent at home; they are unable to discriminate what is peculiarly worthy of their attention, and their time is wasted and their attention distracted, by a diversity of objects, all tending to no useful end, or at any rate, rendering them no greater benefit than they might have obtained, without the expense and trouble of traveling.

With respect, then, to the studies usually attended to previous to graduating, the student can have as great facilities in New York as anywhere; and if, during his preparatory studies, he has also attended to hospital practice, he will graduate with juster notions as to the force of medicine, and its power of overcoming disease, than if he had prepared himself in France. The privilege of dissection, there, is certainly great, when compared with the restrictions on it in England. It is cheaper, too, than with us, but not so much so as to make it any great object. With respect to the study of obstetrics, students are mistaken who expect to have much greater advantages there than in New York. The Hospice de la Maternite, the only institution of any extent, is attended almost entirely by females, and students are wholly excluded from it. The hospital of the Faculty, it is true, has a few beds for lying-in-women, and students have occasionally the privilege of making examinations during the labor. But it should not be forgotten that every student attending the lectures in New York, has an opportunity of managing, every season, several cases for himself. With respect to surgery, I hold that New York is unsurpassed by any place, and except so far as lithotrity and the management of uterine affections are concerned, Paris is infinitely inferior to it. In their management of cutaneous diseases, the French are superior to us, and in their manner of examining medical cases, they surpass us. I do not know, however, that their diagnoses, except in diseases of the chest, are in general superior to our own. But in detecting and discriminating these last diseases, no other school of physicians is able to compete with them.

Before leaving home, then, in order to be able to employ his time to the best advantage, the American student should be familiar, not only with his books, but with the practice of our own physicians and surgeons; and thus knowing what he is able to meet with at home, he can estimate more properly the peculiarities of foreigners.—*U. S. Medical and Surgical Journal, March*, 1836.

2. *Of the refrigerant method, in the treatment of Nymphomania.*—This method, so happily found successful in nervous affections whose principal seat was the circulating centre or the head, has been employed with decided utility, in this painful and debasing malady. Nymphomania, or furor uterinus, is considered a mental alienation, and classed among the monomaniæ. Its symptoms and effects, though described pretty fully in this article, are too well known, at least by students of medicine, to require transcribing here. The most active agent in the cure of this disorder has been found to be, as stated, the cold bath, conjoined with cold affusions over the hypogastric region, and cold uterine and rectal injections. This energetic method must be used gradually, and with caution. Local baths should always take precedence, and the temperature of these should be regulated, according to the susceptibility, from 24° to 20° Reaumur, (86° to 77° Fahrenheit,) down to 14°, 12°, or even 10°, (63°, 59°, 54°, Fahr.) The patient should remain sitting in these ten, fifteen, or twenty minutes, more or less, when she should be carefully wiped dry. These baths should be repeated every day. Injections into the vagina and rectum should be employed at the same time, though the temperature of these should be three or four degrees higher than the baths, inasmuch as the interior temperature is higher, and the parts more sensible than the exterior. Local affusions upon the hypogastric region, have still greater efficacy, especially when made from a height of one or two feet. Two or three minutes suffice for these, at a time. General cold baths complete the refrigerant mode of cure for nymphomania. In ordinary cases, simple local refrigeration suffices for the indication. Two cases are related, one in particular a well authenticated and strong case, of this mournful disease, which this method was speedily successful in removing.—*Ib.*

3. *New therapeutic considerations concerning the employment of the oil of Croton Tiglium.* By D. S. Sandras.—This article is oftentimes, doubtless, brought to us in an impure state, as a specimen examined by the author was found to be *partly soluble in water*, and of which one, two, and even three drops, given in a little sugared water, produced scarcely any stools. Indeed, no article by the name can be employed with certainty, unless we have had some experience with it. Like M. Piedaugel, the author has found that salivation, the sense of bitterness in the throat, of heat, and burning in the chest and along the æsophagus, may be entirely avoided, by rapidly swallowing the oil, without allowing it to touch the membranous parietes of the mouth, and not tasting it. It ought to be remarked, that one drop taken in four pills at intervals of a quarter of an hour, has less efficacy than a erop taken at once in a spoonful of sugared water or mucilage. Great care should be observed in giving the oil of croton, whenever there is any anginal affections, as they are pretty sure to be aggravated. The oil produces very great irritation of the mucous membrane, whenever it comes in contact with it. When properly given, however, it produces with astonishing promptitude, considerable intestinal depletion, frequently occasioning vomiting in doses of one or two drops, and producing eight or ten purgations in subjects before constipated. In obstinate constipations, metallic colics, and once in a case of tenia, the author has seen it act with great promptitude and efficacy. When there is much gastro-intestinal irritation, it should be withheld, but when this is wanted to relieve an irritation in the head, it is of great service. A striking case is related, in which a single drop produced immediately a discharge of several ells of tenia; a second drop, three days after, brought two ells more, and a third drop brought away several fragments more, together with a lumbricoides, more than a foot long, after which the patient recovered.—*Ib.*

4. *Of the rules to be followed in labors connected with a premature birth of the cord.* By Capuron.—The author gives in this paper a review of the various methods which have, from time to time, been proposed by various practitioners, for the relief of this condition of things, so generally fatal to the child. He asks—must we, in this case, act or wait? Are we to disencumber the mother without delay, in order to preserve the life of the infant, or shall we leave all to nature, and confide to it the safety of both? There are two circumstances, one of which we shall generally find on arriving at the scene; either the os uteri is but little dilated, thick and resisting, or it is sufficiently open, thin and soft, to permit, with little effort, the introduction of the hand. In the first case it is evident we cannot and ought not to do any thing. What part ought we then to take? that of expectation, which is most wise and prudent. But while waiting, let us make the most of our time. Instead of remaining idle and tranquil, we should use every means to hasten the dilatation of the uterine neck. Bleed the woman, if she is plethoric; place her in a bath, if she is nervous; give emolient injections in the vagina, or expose the vulva to vaporous fumigations. Guard also the cord from the cold air, but without endeavoring to return it into the uterus, for in the effort, it must suffer compression, and the irritation given to the uterus by so doing, does more harm than good. The cord should, however, if possible, be placed in the position in which it will suffer the least compression, and these vary according to the stage of the labor. In the second condition, where the os uteri is fully dilated, if the labor proceeds well, if the pains succeed each other regularly and at short intervals, if the presentation is good, and the cord possesses warmth, and sensible pulsations, then leave nature to act for hereelf. Those who would hasten to turn the child and apply the forceps, often make two victims to their rashness. If, however, the labor should languish, the uterine contractions be slow, feeble, and ineffectual, we must then revive the labor, even with ergot, at any rate we must hasten the accouchement. Here the hand and the forceps are indicated, according to the position and situation of the infant. If the presentation should be found unnatural, the labor must be terminated in the usual way as speedily as possible. Finally, should every thing be found favorably circumstanced, except the cord, and this should be found void or nearly so of pulsations, the delivery must be accelerated by the hand or forceps, according to the situation of the child.—*Ib.*

5. *Of the treatment of fractures of the lower extremities, without confining the patient to the bed.*—Sir A. Cooper remarks, that in the ordinary method of treating fractures, of the neck of the femur, for instance, some patients, instead of being cured, lose their lives solely from the long confinement to the bed. It produces a general languor in the functions of all the organs, a general diminution of strength, weakness of the digestive organs, leanness, often ulcers from lying, hectic fever, and death. Moreover, we do not now think that a patient must remain immoveable in bed, in order to be cured of a fracture of an inferior limb. M. Mayor, of Lausanne, who, in 1833, called the attention of practitioners in Paris to this point in therapeutics, has proved the possibility of this fact. He has allowed his patients to walk about with crutches, after the tenth day. The apparatus is rendered immovable by a very simple means. We have seen some of M. Mayor's patients promenading their rooms, with the aid of an elbow-chair on castors. The limb was sustained horizontally by means of a shelf, suspended by four strong cords. The means by which the fracture apparatus is rendered immoveable, is this. For the thigh, the whites of thirty eggs; (twenty for the leg,) beat them well in a proper vessel, and add of liquid acetate of lead from four to six ounces, of camphorated spirits the same quantity; mix the whole very exactly. The result is a sort of semi-liquid cream. The apparatus of Scultel, or the separate little bandages, being disposed as ordinary under the limb, the mixture is poured upon their inner surface, and they are then applied to the limb in the ordinary manner. The stuffings and splints are placed above. This mixture dries in two or three days, and forms a sort of mastic, as hard as a board. The limb is enclosed as if in a box, during the ordinary time of the consolidation of fractures. When it is to be removed, the patient must be put into a warm bath. In treating fractures of the upper extremities, it is sufficient to immerse the bandage in the mixture.—*Ib.*

6. *Method of forming an artificial Pupil without injury to the chrystalline Lens or its Capsule.*—The *Medical Quarterly Review* for Jan. 1835, contains an interesting paper by Mr. Tyrrell, Surgeon to the London Ophthalmic Infirmary, on this subject. "There has not been any plan devised," according to Mr. T., "by which the surgeon can form an artificial pupil, without at the same time injuring the chrystalline body; so that, if it were previously sound, it would be rendered opaque by an operation for forming the artificial pupil, and consequently its removal would be necessary, to make the artificial pupil serviceable

"The loss of this highly important structure is not the only evil resulting from injury to it, for such injury often produces consequences fatal to the formation of the new pupil itself, and is sometimes destructive to vision altogether.

"Thus the opaque lens, or some fragments of it, by pressing on the iris, often create and maintain an inflammatory action in the iris, under which the artificial opening closes; or the inflammation thus excited extends to the deeper seated tissues, (as the choroid and retina,) and produces such changes as destroy their functions, and occasion organic amaurosis."

The following are the cases to which this operation is applicable, and Mr. Tyrrell's mode of performing it:

"An aperture penetrating the cornea, whether created by wound or by ulceration, immediately permits the escape of the aqueous fluid, and is usually followed by a prolapse of the iris; and if the aperture be large, so much of the iris is sometimes protruded that the pupil becomes exceedingly diminished or destroyed; and in the reparation of the mischief, the iris becomes adherent to the cornea, and embedded in the cicatrix, constituting *synichia anterior*. In the cases in which the pupil is nearly destroyed the remaining part is often useless, in consequence of the portion of the cornea which covers it becoming opaque in the process, by which the injury to the cornea is repaired.

"When the opacity of the cornea is irremediable, and the remaining part of the pupil is so small that the influence of belladonna cannot increase it, an operation is as necessary to restore vision, as in those cases in which the pupil has been entirely destroyed from a similar cause.

"When under these circumstances, one-third or more of the cornea retains its transparency and natural character, and the subjacent portion of the iris is of healthy aspect, the patient will probably have a distinct perception of light, and a perfect result may then be anticipated from operation; but when less than one-fourth of the cornea is free from disease, a less favorable termination to operation may be expected: we may hope to restore useful, but not perfect vision.

"When the iris is discolored or dull, it has been inflamed, and it may be adherent to the anterior capsule of the lens, and the capsule itself be opaque. The evidence of previous inflammation of the iris, therefore, renders the prognosis of the case, as regards the result of an operation, very doubtful; but it should not deter the surgeon from its performance, for I have often restored good or useful vision when I have had little prospect of success.

"If the patient can distinguish light from darkness, I always consider the operation warrantable, as affording a chance of benefit, even when the features of the case are otherwise most unfavorable.

"The operation is then applicable to all cases in which the pupil has been entirely destroyed, or so nearly so as to be useless, in consequence of prolapse of the iris, provided that a portion of the cornea remains clear, and the patient has a perception of light.

"It sometimes happens that the natural pupil becomes obscured, and rendered almost useless, by the formation of a dense and large central opacity of the cornea, as the result of cicatrization of an ulcer, or from the effect of an escharotic, and the opacity is permanent. In such a case vision may be improved or made good, by altering the position of the original pupil, which can be readily effected by the operation I am about to describe.

"The instruments required in the performance of the operation are, first, a knife or needle, to make an aperture in the cornea of sufficient size to admit of the introduction of a hook. As it is desirable that the opening in the cornea should not be much larger than is sufficient to admit the hook, I prefer a needle of a diameter rather larger than the hook to a knife.

"Secondly, a hook, the extremity of the curved part of which must be perfectly smooth.

"In forming an artificial pupil, on the nasal side, I have found it necessary to have the stem of the instrument bent nearly at a right angle, to enable me to avoid the nose.

"Thirdly, a fine pair of scissors, to cut off a portion of the iris to be brought out of the opening in the cornea by the hook.

"Fourthly, a pair of fine dissecting forceps.

"The operator has not usually much choice of position for making the artificial pupil, as it must of course be formed under the transparent part of the cornea, which is frequently very limited; but he should at all times, as far as circumstances will admit, form it towards the inferior part of the cornea, vision downwards being of much greater service than upwards.

"During the operation I prefer having the patient recumbent, with the head slightly raised, and with the light falling rather obliquely on the face. Then seating myself at the head of the patient, so that his head rests against the lower part of my sternum, I can command the superior eyelid of either eye, and hardly require the aid of an assistant.

"In elevating and fixing the superior palpebra, the fore finger should be placed near the centre of its free margin, so that the extremity of the finger touches the surface of the globe, and the cilia are pressed outwards: by very moderate force the lid can then be elevated, but, in effecting this, the fore finger must not only press against the eyebrow or supercilliary ridge, but also slightly against the surface of the globe, or the eyelid will probably avert. At the same time the point of the middle finger should be placed on the globe, near the inner canthus.

"The left hand should be used to the right eye, and the right hand to the left eye, when the surgeon occupies the position described; but if not ambidexter, he must change his position, and employ an assistant when operating upon the left eye.

"By well-regulated pressure with the points of the two fingers, the surgeon can in a great measure command the motions of the globe, in most instances, and thereby facilitate the operation.

"The superior lid being elevated and fixed, and the globe under command, the needle is to be passed through the cornea, close to its junction with the sclerotic, at the point previously determined upon; it should freely enter the anterior chamber.

"On withdrawing the instrument, a part or the whole of the acqueous fluid usually escapes; and, if the iris has been previously free and unaffected, (as in the simple case of dense central opacity of the cornea,) a small part of it may prolapse; and I always endeavor, by firm pressure with the fingers placed on the globe, to effect such a protrusion.

"A prolapse of the iris, however trifling, immediately occasions an alteration in the figure and in the position of the pupil; and thus, the puncture of the cornea being followed by a spontaneous protrusion of the iris, sometimes affects all that is desired in such a case, by bringing the pupil under a transparent part of the cornea.

"If under these circumstances the prolapse of the iris be not sufficient, the projecting part should be seized by a fine pair of forceps, and drawn from the wound, until sufficient has been pulled out to effect the desired change in the position of the pupil.

"Supposing that the iris does not prolapse when the anterior chamber has been opened, in the case of dense central opacity, or when the operation is resorted to, in consequence of the original pupil being nearly destroyed, the further steps of the operator will be similar.

"The patient being allowed a few moments rest after the use of the needle, the upper eyelid should be again elevated as before, and the hook be carefully passed through the opening effected by the needle into the anterior chamber, and carried on until the point enters the pupil. In passing the hook, the curved part should be kept towards the cornea, until the extremity has entered the pupil, when the instrument should be rotated, so that the hook may receive the free margin of the iris.

"The iris being caught by the hook, the instrument should be steadily withdrawn, and when the curved part arrives at the aperture in the cornea, if it is arrested, the instrument must be again rotated, to turn the curved part towards the cornea, otherwise there may be some difficulty in getting it out, in consequence of the extreme part of the hook catching against the inferior edge of the aperture. The hook brings out a portion of the iris, and produces an immediate alteration in the position of the natural pupil, or an enlargement of that previously diminished by disease, and by gradually withdrawing the instrument from the aperture, a further portion of the iris may be generally pulled out, until the desired effect on the pupil is produced. The protruding portion of the membrane may then be removed close to the aperture in the cornea by the scissors. If the hook tears through the part of the iris which is brought out of the anterior chamber by it, before a sufficient portion has been drawn out, the protruded part should be seized with the forceps, and a further portion withdrawn and cut off.

"Supposing that the original pupil has been entirely destroyed, it is necessary to make a small aperture in the iris as well as in the cornea, for the passage of the hook; and this is the only respect in which the operation in such a case would differ from that described. The opening in the iris should be made by the same instrument by which the cornea is penetrated; and, if a needle be employed, the opening in the iris can be effected without increasing the size of that in the cornea. After, therefore, the needle has entered the anterior chamber, the point should be very carefully directed to the part at which the iris and cornea are adherent, and passed through the former close at its junction with the latter; a very small opening suffices. In puncturing the iris the operator must direct the point of the needle to the cornea, for there is usually in these cases so little space of posterior chamber, that the capsule of the lens may be easily injured if the point of the needle be passed at all backwards. The subsequent steps of the operation in such a case should be as I have already described."—*Ib.*

7. *Amaurosis from Metallic Colic.—The Archives Generales* for May, 1834, contains a memoir on Amaurosis from lead. The author, M. Duplay, endeavors to establish the differences and analogies between amaurosis from lead, and the amaurosis which is found in some instances to accompany a nervous colic, the symptoms of which strongly resemble the lead colic; though they clearly do not depend upon the same cause. To this end, M. Duplay first gives cases from former Nos. of the Journal in which he writes, from Andral's Clinique, and from his own experience, illustrative of amaurosis from lead colic; and others quoted from Felix Plater, Henry Smetius, Hildanus, Lucas Stoerck, &c., descriptive of the attack of amaurosis in nervous colic; a case, however, which he observes is sufficiently rare. Reasoning from these he comes to the following conclusions:

1. The amaurosis which succeeds metallic colic, or nervous colics resembling it, is peculiar in the sudden and almost instantaneous manner of its attack; the patients losing all vision, and becoming incapable of distinguishing night from day in a few hours.

2. It commonly shows itself after several attacks of colic, but may occur in the first, just as do other derangements of inervation in persons having those seizures.

3. In the majority of those seized with amaurosis, this system is preceded by other nervous disorders, as pain of the arms, cramps of legs and belly, &c.; as also by palsy of the fingers, and more commonly still by epileptiform attacks and delirium. At other times it appears alone, and is only succeeded after some time by nervous symptoms.

4. When the patient is in a complete state of blindness, the pupil, on examination is found to be considerably dilated, and utterly immovable. M. Duplay says he has not recognized the character of metallic amaurosis mentioned by Heller, namely, a turgescence of the vessels of the conjunctives and sclerotica, with a sense of fullness of the eye-ball.

5. The amaurosis from lead acquires its greatest intensity in a few hours. In other varieties of amaurosis, this sudden obliteration of sight is rare, the falling off being more gradual.

6. The disease in question is for the most part of short duration, varying from a few hours to some months. The average term seems to be five or six days. In one of the cases it lasted two months. In another, an amaurosis in the nervous colic, related by Plater, become permanent.

7. The number of relapses of colic does not appear to influence the extent of the amaurosis; it often quickly disappears in persons that have been repeatedly attacked with lead colic, and, on the other hand, is often obstinate in the first attack.

8. In most of the cases the amaurosis has disappeared under the treatment of metallic colic; as the symptoms of the latter diminished, the sight began to return.

The amaurosis from nervous colic is best treated with the purgatives, according to M. Duplay.—*Amer. Jour. of the Med. Sciences.*

8. *Pommade for the cure of enlarged tonsils.*—Dr. Cerchiari, in a communication in the *Bull. delle Scienze Med. de Bologne,* May, 1835, extols the efficacy of the following ointment, in the cure of enlarged tonsils, caused by repeated attacks of inflammation: ℞ Iodin. pur. ℈j. Ung: Ros. ℥j. m. To be applied to the tonsils morning and evening, by means of a small brush. By the end of two months these glands will, he asserts, under this application, return to their normal size. It is necessary that the inflammation should be entirely subdued before recourse is had to this ointment.—*Journ. de Conn. Med. Prac., Aug.* 1835.—*Ib.*

9. *Hemoptisis cured by Kreosote.*—Dr. Sentine has reported in the *Gazetta di Therapeutica de Verone,* for March, 1834, a case of profuse hemoptisis, recurring at short intervals, and occurring in a man forty years of age, in which a persevering antiphlogistic treatment, general and local bleeding, cutaneous irritation, and astringents, had been employed without success. Dr. S. administered the following potion: ℞. Muc. Gum: Arab: ℥iij. Kreosote gttv, Syr.: Althææ: ℥j. A spoonful of this mixture was given every three hours, and afterwards every two hours. After the first two doses, the orgasm and pain of the chest diminished, the respiration became freer. The quantity of kreosote employed was one drachm.—*Ib.*

10. *On Hydrocele of the neck with cases and observations.* By James O'Beirne, M. D.—"It is now nearly twenty years since Professor Maunoir, of Geneva, described a disease to which he gave the name of hydrocele of the neck, and which, although essentially different in its nature, and requiring a very different mode of treatment, bears such a resemblance to bronchocele or goitre, that it has been constantly confounded with the latter disease, and treated accordingly. The manuscript memoir in which he described this disease was read at the Royal Institute of France in 1815, and afterwards transferred to the Academy of Natural Sciences, by which body the late celebrated Baron Percy was selected to report upon its merits. It was not, however, until April, 1817, that the Baron presented his report, which proved highly unfavorable to Professor Maunoir's opinions and practice. In 1825 the latter published, for the first time, his memoir, with the whole of the unfavorable report made thereon, and a most able and satisfactory defence of his peculiar views on the subject.* But it would appear that, as too often happens, the authority of a great

*Memoires sur les Amputations, l'Hydrocele du Cou, et l'Organization de l'Iris. Par J. P. Maunoir, aine, Prof. D. C. Geneve et Paris, 1825.

name, aided by bold and specious objections, proved more powerful than either the strongest facts or arguments; for, after considerable research, I have failed in finding even the slightest notice of this memoir in any subsequent French or English work. So little, indeed, does it appear to be known in both countries, that Delpech* and Lawrence,† who, between them, have related three cases, which appear to have been examples of the disease, not only make no allusion to it, but by employing incision in the treatment of three cases, would seem to show that they were unacquainted with its existence; for it is only natural to presume, that if they had known the equally certain, and less dangerous and disfiguring mode of treatment by seton, so successfully adopted by Maunoir, they would have given it the preference.

About four years ago the three memoirs to which I have already referred came accidentally into my possession, and the singularity of its title induced me to read the "Sur l'Hydrocele du Cou." Since that time, accident again favored me by enabling me to observe three striking examples of the disease, all of which displayed the utter fallacy of Baron Percy's objections. According as they presented themselves, accurate notes and drawings of these cases were taken, with a view to publish them, at some future day, and give such a general account both of the memoir in question and the whole subject as might prove acceptable to the profession. That time is now come, and I trust, that, aided by the two annexed lithographic plates, I shall be enabled to carry my intentions into effect.

According to Professor Maunoir, the disease has been often observed without its true nature being known; as may be seen in treatises on tumors, and from one detailed by Heister, and three cases quoted by Plouquet. He declares also, that all the cases of it which he has seen, had been confounded with and treated as goitre, by numerous members of the profession. The disease consists in the formation of serous cysts, commencing very small at some point of the side of the neck, and gradually increasing for several years, to such a size as to occupy the whole of the front and of one side of the neck, and seriously to impede respiration, deglutition and speech.

The tumor so formed conveys to the touch a distinct sense of fluctuation, and contains a fluid of either a limish, or a dark coffee color, and coagulable by heat. In the great majority of instances, it exists independently of any enlargement of the thyroid gland: and, in his fourth case, it was situated behind the angle of the lower jaw, and, of course, quite removed from this gland. But he has, in two instances, observed the contrary, and the second of his cases, in which the gland enlarged and indurated formed one-eighth of the whole tumor, is an example of this complication.

With respect to the treatment of this disease, the learned Professor's opinions and practice are these: "Although," he says, "there may be great affinity between encysted tumors in the neck, and hydrocele of the tunica vaginalis, yet it appears to me that, in hydrocele of the neck, the cyst is more dense, and more difficult to be excited to adhesive inflammation. Accordingly, its treatment should not be directed by analogy, and it is not proper to have recourse to the cure

*Chirugie Clinique de Montpelier, t. ii. p. 79—87.

†London Medico-Chirurgical Trans. vol. xvii. p. 44, et seq.

by injection, although it seems, at a first view, to be the best. I wished to try it, and have been obliged to renounce it as a bad plan, and not one free from danger. An injection, which is not very stimulating, will effect nothing, or almost nothing, on a very thick, and in general, old cyst. If a very active injection be employed, it will cause great pain, and give rise to very alarming spasmodic symptoms. Moreover, I have to observe, that sometimes enlargement of the thyroid gland complicates the treatment. In that case, the object is not merely to produce adhesion of the walls of the sac; it will be necessary to employ a mode of cure by which we may succeed at the same time in resolving this gland, when it projects into the tumor, as I have seen in two patients." As to laying open the tumor by incisions, as practiced by Heister, or extirpation of the whole or of only a part of the cyst, he condemns these operations as being serious, difficult, and calculated to prolong a cure, by producing a large wound, and one of a kind very slow in cicatrizing. In short, the treatment which he has been led to adopt and recommend, consists in puncturing the tumor, and, after evacuating its contents, passing a seton through it in the direction of its longest diameter. By this plan a fresh accumulation of fluid is prevented, the adhesion of the walls of the cyst is insured, and the thyroid gland when it happens to be enlarged, is gradually reduced to its natural size.

He relates three cases, all of which are so generally interesting, that I shall here give them in a comparatively abridged form.

Case I. A washer-woman named Martin, aged 49, still menstruating, with a spherical tumor on the front and left side of the neck, as large as an infant's head, presented the first example of the disease that the Professor had seen, read, or heard of. Originally this tumor had been very small, but increased in quite an insensible manner. It did not force the head to incline to the left, but to the right side, and formed a sort of cushion for her head to rest upon. She had taken burnt sponge, and many other boasted remedies for goitre, but without any benefit. Difficulty of breathing and swallowing came on, and increased in proportion to the growth of the tumor. One day, while washing at the river side, she threw up a very great quantity of blood, fainted, and was supposed for some moments to be dead. The hæmoptysis and oppression continuing, and the swelling being felt to contain a fluid, a trochar was passed into the most prominent and fluctuating part of the tumor, and gave exit to a pint and a half of a deep brown liquid, which coadjulated by the application of heat. Complete relief ensued. On the following day, the swelling had returned to its former size; but fluctuation was less manifest for infiltration had taken place between the tumor and the skin.

At the end of fifteen days this infiltration had disappeared, and the cyst was punctured by a trochar, and after being emptied, filled with warm red wine and a small portion of alcohol. This injection, although retained but for a few moments, caused great pain and suffering. Swelling, redness, trismus, and increasing pain, on the following day: leeches, poultices, aperient medicines, and opium, ordered. An abscess, external to the cyst, opened and treated in the ordinary way, until it healed. A third puncture made in the upper part of the cyst by a sharp-pointed bistoury, and giving exit to as considerable a quantity of fluid as at the second. A button-pointed probe was then introduced into the opening, and passed until it became prominent at the most inferior part of the tumor; the point of the probe then cut upon, and the instrument withdrawn, leaving in its place a single thread. This thread frequently renewed: no accumulation of fluid. A seton of ravelled linen passed, and caused abundant suppuration. This seton continued for six weeks, and then removed by the patient, on account of interfering with her ordinary occupations. Both openings fistulous for some months; the upper first closed; and in the year 1813, when she was 63 years of age, her neck was very slender, and her health robust.

Case II. Monsieur C., of Vevay, aged 40, had for many years a tumor situated on the front and right side of the neck. This tumor extended from the chin and lower jaw to the sternum and clavicle; and in the greater part of its extent, there was a manifest sense of fluctuation, but points corresponding to the thyroid gland appeared to be hard and prominent. The swelling increased daily, became fatiguing from its weight, and ultimately caused difficulty of respiration and speech, and occasionally attacks in which he seemed to be on the point of expiring. A puncture made into the upper and left portion of the tumor, and a pint of limpid, amber-colored, and perfectly inodorous fluid evacuated. This evacuation reduced the tumor to one-eighth of its size, the remaining portion being formed by the thyroid gland in an enlarged and indurated state. A blunt probe now introduced into the opening in the sac, and carried down to the inferior and anterior portions of the tumor; the point of the probe cut upon, and a single thread passed, in the usual way, as a seton. Great freedom of respiration and in moving the head, instantly followed the complete evacuation of the tumor. Next day, a fresh accumulation of fluid, but much less in quantity and of a fetid, sanious kind; some fever; stomach deranged. Hippo, followed by infusion of bark, and Spa and Seltzer waters employed, and restored the patient to his ordinary calm state. Pieces of linen gradually increased in size, and smeared with simple digestive ointment, introduced as setons; injections of plain and hydrosulphurated water, and decoction of bark, with honey thrown into the sac.

Discharge less in quantity, and more purulent; the extent of the cavity greatly contracted; and the thyroid gland diminished in size. In a few months the patient's health was completely restored, and his neck became of its natural size.

Case III. Mademoiselle T. D., aged 20, having for many years a large tumor on the front, and a little to the right side of the neck, had been subjected to all the known modes of treating goitre. This tumor was of enormous size, and consisted in a great degree of fluid. The least movement brought on cough, and attacks of suffocation. Her parents and friends refused to permit a seton to be passed, but a puncture with a trochar was made in the most depending part, and a cupful of fluid, resembling infusion of coffee, was drawn off. The canula was then withdrawn, with a view of retaining the rest of the fluid, and enabling a second puncture to be made and a seton to be passed. The tumor was very little diminished; the wound was then covered with adhesive plaster, and a roller applied with moderate firmness. After passing some hours in a very quiet state, she indulged too freely at dinner, and in the evening felt oppressed in her breathing, and the tumor became quite black. It was evident, in fact, that the contents of the sac had passed into the subcutaneos cellular membrane. She passed the night badly, and could scarcely swallow a few drops of an anodyne draught. In the morning great difficulty of respiration, and total incapability of swallowing; the parts surrounding the tumor so swelled that the neck was raised to the level of the chin and lower jaw, with which it seemed to form one continued pillar. The whole of the upper part of the thorax was also infiltrated, and the alteration of the voice and dispnœa were such as to lead to the belief that the effervesced fluid had penetrated into the internal cellular tissue of the trachea. In the course of the day, however, all these symptoms gradually diminished in severity, and the swelling was considerably reduced towards evening. She passed a good night, and on the following morning deglution and respiration were free. On the fourth day from the operation, the original tumor was diminished by one-half, the infiltration and black color of the skin had disappeared, and the patient was in excellent health.

On the 30th of January, 1812, that is, after about six weeks had elapsed, the tumor was as large and as distressing as ever. A hydrocele trochar, with a flat elastic canula, was passed into its most depending part, and two pints of a dark brown fluid, coagulable by heat, were discharged. On emptying the tumor, the thyroid gland was found moderately enlarged. A blunt probe, armed with a single thread, introduced through the canula, made prominent at the upper part of the cyst, and there cut upon until it could be withdrawn, and the thread left as a seton. For some days nervous symptoms appeared. The two little incisions contracted so much, that the thread could not be moved backwards and forwards but with great difficulty, and such as to create a suspicion of its being lodged in the tissues of the walls of the cyst, which it had cut in gliding, and of having thus left the cavity of the tumor. The silk thread withdrawn, at the instance of her parents, and in order that a fresh accumulation might permit a puncture to be made by a bistoury, (instead of the trochar which had been found so ill-suited,) and enable a cotton wick to be passed as a seton. The tumor soon regained its formsr size, and the oppression returned. The necessity of this operation repeatedly urged, but as often delayed from some frivolous pretext. The Professor sent for in great haste, on the 16th of April, 1812, and found her with complete loss of sense and motion, slow and stertorious breathing, cold extremities. dilated pupils, and no pulse. No person being at hand to assist in the proposed operation, the tumor was punctured by a hydrocele trochar, and a pint of dark brown fluid discharged. Immediately pulse, respiration, and in short, animation were restored; but permission to pass a seton could not be obtained. On the 7th of May, the size of the tumor required that it should be again punctured. On the 24th of June, she complained of violent pains in the head, great suffering and oppression. Another puncture made in the swelling, and a quantity of fluid mixed with purulent matter, discharged. 25th, pains returned; astringent applications; increased enlargement of the neck; distress and oppression alarming. Six leeches applied, and the patient well purged with castor oil, without any relief. 27th, tumor punctured, and a lesser quantity of fluid, but more mixed with pus, discharged.

21st of July, symptoms severe, and increasing so much in violence, as to require another puncture, which was rendered difficult by the thickness which the infiltrated cellular membrane had acquired, and, consequently, the increased depth at which the cyst was placed. A silk thread, and, subsequently, a large seton inserted; abundant fetid suppuration; gradual contraction of the sac; an abscess formed and opened at the inferior and lateral part of the neck; a fistulous opening for some months at this point, and at length healed by an injection of a weak solution of sulphate of copper. Seton removed; tumor completely dispersed; and recovery perfect in all respects.—*Dublin Jour. of Med. and Chemical Science.—Ib.*

11. *Radical cure of Variocele.*—Dr. Frick, of Hamburgh, has cured three patients laboring under variocele, by simply passing a needle and thread through the dilated veins, and allowing the thread to remain for twenty-four or forty-eight hours, according to the degree of reaction produced. He recommends the method as being as safe as it is simple.—*Gazette Med. de Paris.—Ib.*

12. *Goitre—extirpation, cure.*—Professor Graefe, of Berlin, in 1833, performed the operation for the extirpation of groitre in two cases, with success.

Case I. A young man, 22 years of age, had a tumor the size of a goose's egg in the anterior and middle part of the neck, which occasioned extreme difficulty of deglutition and respiration. These symptoms, so little in accordance with the small size of the tumor, rendered it probable that the latter adhered closely to the anterior part of the larynx and trachea, which circumstance M. Graefe was careful to keep in view during the operation. An incision was made through the skin, commencing a finger's breadth above the superior margin of the thyroid cartilage, and extending down the median line of the neck to the top of the sternum. The subcutaneous and sternomastoid muscles were then drawn to the right and left, which exposed the tumor, presenting a shining aspect. The surrounding parts were detached with the finger, and a bistoury, and some arteries were tied. The tumor was now found to adhere closely to the larynx and trachea, without the intervention of any cellular substance. The excision of the goitre was performed with the greatest caution by small strokes of the knife, and the portion of it which adhered to the air tube was not removed. Only eight arteries were tied during the operation. The wound was filled with lint—union by the first intention being avoided, in order that the still adherent part of the tumor might be discharged by suppuration, which accordingly took place. The lips of the wound were afterwards placed in accurate apposition, and the cure was complete at the end of six weeks.

Case II. A young woman, twenty-five years of age, of a delicate constitution, had been affected from her infancy with a goitre which was divided into three very distinct lobules. The immense size of the tumor precluded its entire removal at one operation; it was therefore determined to begin with the middle lobule, which was the largest, and appeared to be the nucleus of the morbid growth. The operation was conducted as in the preceding case, except that the tumor being attached to the larynx and trachea only by loose cellular tissue, there was no necessity for leaving any part of it adherent. The wound was united by adhesive straps, and was entirely cicatrized in six weeks. The lateral lobes, instead of enlarging, as there was reason to fear, diminished considerably, confirming the opinion of M. Graefe, that the middle lobe formed the nucleus of the tumor. Perhaps, also, the inflammation consequent on the operation, and the obliteration of the vessels that were tied, contributed to the absorption of the remaining lobules.—*Ryon's Lond. Med. & Surg. Journal, 4th April,* 1835.—*Ib.*

13. *On the removal of Sequestra without an operation.*—Dr. Bouget has published a new plan for the removal of sequestra without an operation, in the *Journal de la Societe de Medecine de Bourdeaux*, in an article entitled, "Souvenirs de la Clinique de Delpech."

M. Delpech, discouraged at the unfortunate results in several cases of necrosis of the tibia, turned his attention to measures which might remove the sequestrum without having recourse to the painful operation which is generally necessary. In this search he was successful, for he found that, by means of diluted sulphuric acid, he could destroy the phosphate of lime in the bone to be removed, which is then reduced to its geletinous parenchyma, and can be easily taken away with the common dressing forceps.

Delpech first employed this application in the year 1814. At this period, the wounded at the battles of Orthes and Toulouse, flocked in such numbers to Montpelier, that the Hospital St. Eloi was soon crowded, and a supplementary one was formed, at the head of which was placed M. C. Fages, since so well known by his valuable lectures on external pathology. Hospital gangrene soon appeared in both hospitals, and caused such extensive ravages that the majority of the amputations terminated fatally; even in those cases which were the most successful, a greater or smaller portion of the bone was left exposed by the destruction of the soft parts. A young man, who had undergone amputation of the arm, and had twice suffered from hospital gangrene, which had been with difficulty arrested, had the humerus projecting about an inch and a half beyond the flesh. According to the ordinary treatment the sequestrum would not separate perhaps for months, but it happened far otherwise under M. Delpech's directions. He caused the external surface of the bone to be covered with a pledget of lint, soaked in dilute sulphuric acid, and a wad of the same, equally wetted, to be passed in the medullary canal, whence the reticular apparatus had been previously removed; at the end of twenty-four hours the portion of denuded bone was so softened that it could be easily detached: ten days after the extremity of the bone was covered with fleshy granulations, and a complete cure was speedily accomplished.

In the year 1816 a man entered the clinical ward, having a necrosis which extended through the whole length of the tibia. Although he evidently possessed a good constitution, and was apparently capable of undergoing a serious operation, M. Delpech determined to have recourse to the proceeding which had proved successful in the previous instance. He destroyed the soft parts at the upper part of the leg by means of the potassa fusa, and when the eschar, which was about the size of a crown-piece, had sloughed, he applied a pledget of lint, soaked in the dilute sulphuric acid, to the bone; after two or three dressings, renewed every five or six hours, it became soft enough to be taken away by dressing forceps. This being effected, the application of the potassa, followed by the acid, was made lower down; the sequestrum was exposed to the extent of five or six inches in length, and an inch and a half in width; it was then extracted with the greatest ease. It was more than six inches long, and constituted nearly two-thirds of a cylinder. The patient left the hospital quite well one month after his admission.

From that time until 1822, when I left Montpelier, adds M. Bouget, I have seen M. Delpech constantly have recourse to this plan of treatment, both at the hospital and in private practice, and always with success. I have also used it myself with advantage in a case of necrosis of the tibia in a child.—*Ib.*

14. *On the effects of Poisons on the Animal System.*—A report from Dr. Roupell was read at the Dublin Meeting of the British Association, the object of which was to show the effects produced by poisons introduced into the circulatory system, and the affinity they appear to exercise for the component elements of different parts of the body. Several plates were exhibited, illustrating the results.

Plate 1 represented the stomach and intestinal canal of a dog, poisoned by *arsenic*. An ounce of the saturated solution of arsenic, made by boiling arsenious acid, and allowing it to cool, was injected into the femoral vein of a dog. In three minutes afterwards, the animal became sick, and made an attempt to vomit; his breathing also became very much hurried. In ten minutes more, great intestinal movements appeared to be going on, and the abdominal muscles were forcibly contracted; in twenty-five, vomiting took place, followed by paralysis of the hind legs; in thirty-five the animal died. The body, when examined shortly afterwards, was found rigid, the blood fluid, the lungs stuffed with mucous, but not inflamed. The peritoneum was rough, and had lost its shining appearance; the stomach presented the hour-glass contraction, and was found to contain an ounce of tough mucus. The great end was much inflamed, the lesser differed very little from its healthy state. The large intestine was free from disease, and contained solid fæcal matter. The chief alteration was in the small intestine, which was extensively inflamed, and covered with a layer of tough mucus, tinged with blood. There was no change in the mucous membrane of the trachea or bronchi; or in the lining of the heart, veins, or arteries. The chief points of interest connected with this experiment are the absence of inflammation in those parts with which the poison came in contact, and the circumstance of its being restricted, almost exclusively, to the intestinal canal.

Plate 2 represented the stomach of another dog, poisoned in the same way. The subject of this experiment was a strong animal, and the solution of arsenic which was employed had been filtered. The same quantity, however, was administered as in the former case, and the poison was injected into the femoral vein. Shortly after the operation, vomiting took place; in twelve minutes solid fæces were passed from the bowels, followed by tenesmus. In 35 minutes, vomiting, cramps, and dysenteric symptoms occurred, which continued with more or less severity, and in about two hours the animal died. The lungs were injected, but not inflamed. The stomach and intestines were universally inflamed; the former contained about four ounces of frothy mucus, the latter, throughout its entire length, a bloody secretion. The mucous membranes of the rest of the body were redder than natural, but no change could be detected in the lining membrane of the venous or arterial systems. In this instance, the longer interval between the injection of the poison and the death of the animal gave time for a greater extension of inflammation.

Several experiments were made with smaller quantities of the arsenical solution, but without any fatal result. Half an ounce, thrown into the femoral vein of a strong dog, appeared to produce but little inconvenience. Half an ounce of the *liquor hydrargyri oxymuriatis* was injected into the veins without producing any appreciable result. An ounce, (which contains half a grain of the corrosive sublimate,) produced dysentery and considerable distress, but not death.

The next trials were made with *tartar emetic;* the preparation employed was the vinum antimonialis. An ounce of this was thrown into the saphena vein of an active terrier. The first and almost immediate effect of this was to produce symptoms resembling intoxication—a circumstance which may be attributed to the quantity of alcohol contained in the preparation employed. The animal was able to stand and run, but reeled about and tottered in his gait. On being visited some hours afterwards, it was found dead; from the appearance of its jaws, vomiting seemed to have taken place. The brain was found natural in appearance, and the intestinal canal presented nothing different from the normal state; the chief alteration appeared to have occurred in the stomach, which exhibited signs of intense vascularity, particularly at its greater end.

Several other experiments were made, but of less interest: when a solution of metal in strong acid was employed for the purposes of injection, death took place rapidly, and the mucous membrane presented a marked red appearance. Dr. Roupell had been able to satisfy himself as to how far such changes in the intestinal canal are to be attributed to the compound, or to the effect of the simple acid, which in itself would coagulate the blood, or greatly predispose to that condition. The injection of a solution of *kreosote*, a substance which appears to possess the greatest power in this way, has no influence on the intestinal canal. A drachm of this substance, mixed with water, produces no effect; but when injected pure, death has been the result. The appearances seen on dissection in this case, were confined to the lungs, which were black and gorged with blood that appeared to consist of minute granules, mixed with a fluid of inky blackness.

Dr. Roupell concludes, that any attempts at explaining the effects of the foregoing experiments, must, in the present state of animal chemistry, be merely conjectural. How far poisonous substances prove irritant by their chemical agency, or by exciting in certain parts the peculiar susceptibility to inflammatory action, it is not easy to determine, but it must be admitted that something more than mere contact is required in those cases where irritants applied to the surface, or thrown into the veins, provoke inflammation of the intestinal canal. Whether it be, that the system is on its guard against those substances which tend to increase the coagulation of blood, must be made a matter for future investigation; but certain it is, that substances endowed with this property seem to have a great tendency to excite the inflammatory condition. It is a curious fact also, as connected with this point, that the coagulation of the blood becomes diminished under such circumstances, or in other words, that coagulation goes on more slowly in an inflamed state of the blood.—*Dublin Journal of Medical Science, Sept.* 1835.—*Ib.*

15. *Case of Poisoning with Arsenic.*—Dr. J. A. Symonds has recorded in the 3d vol. of the *Transactions of the Provincial Medical and Surgical Association*, a most interesting account of the examination and appearances of a corpse fourteen months after death, and of the detection of poisoning by arsenic. The subject of this case was a Mrs. Clara Ann Smith, deceased, who had been interred on the 28th Oct. 1833, and whose relatives having had reason to suspect that her death had been caused by poison, procured an order for the exhumation of the body. This order was executed with the proper precautions, on the 24th Dec. 1834, that is about fourteen months from the period of burial. Dr. Riley, Messrs. Kelson and Herapath, and Dr. Symonds were officially engaged; the two first to conduct the autopsy, the third named to receive the stomach and intestines, with their contents, for chemical examination, and the last mentioned gentleman to witness the proceedings.

"The grave was situated upon the south side of the church, and was about six feet in depth. The soil was a rich and somewhat humid mould, consisting of the red loam which generally covers the new red sand stone in this district, mixed with a large proportion of organic matter. There was no water in the grave. The coffin, having been raised by ropes, was placed upon a flat tomb stone hard by, as there was no convenient room in the neighborhood for making the examination: a circumstance which we should have less regretted had the weather been less inclement. The thermometer stood at two or three degrees below the freezing point. The wood was elm, about two-thirds of an inch in thickness, and bore no marks of decay; but there was a split along the middle of the lid for more than three-quarters of its length: an accident which, I am informed, very commonly takes place, from the weight of the superincumbent earth. The screws having been removed without difficulty, the removal of the lid exposed a corpse invested with a common shroud. A considerable quantity of dark-coloured water, which doubtless had entered by the fissure in the lid, was found collected at the bottom of the coffin, and was in contact with the back of the trunk and head of the corpse, to which parts it was then confined by the inclination of the stone on which the coffin was resting. The flannel lining of the coffin, where it was visible, was quite entire. The shroud, likewise, presented no hole or rent in the upper part, but gave way very readily when pulled; and the shift under the shroud was also in a state of integrity, though softened. Both these coverings had a soddened appearance. A quantity of fine mould lay upon the shroud along the chest and abdomen, having evidently fallen in through the rift in the lid. The head was covered with the remains of a cap, perfect anteriorly, but much decayed where it had been in contact with the water. The face of the corpse was shrunken, and of a dingy yellow color; the nose depressed; the orbits sunk; the cheeks collapsed and wrinkled; and the mouth looked as if open, in consequence of the retraction of the lips. The appearance, on the whole, was not very unlike what would be presented by a wet bladder strained over a skull. The integuments of the trunk had a dull white aspect. The abdomen was considerably flattened, but the thorax had maintained its usual convexity. An incision was made on each side, through the integuments and cartilages of the chest, and continued through the abdominal parietes, and the flap was turned down over the pubes.

"*Abdomen.*—The state of the alimentary tube excited the surprise of the by-standers, by its remarkable degree of preservation; every part being almost as distinct as if the inspection had been made at a very short period after death. The peritoneum was smooth, but rather less glistening than usual, and of a duller white. The intestinal canal appeared to contain neither fluid nor gas, and some of the convolutions were matted together. The omentum was firmer than in a recent body. The liver was shrunk to a fourth or fifth of its volume, and was of a very dark colour. The spleen was black, pretty firm, and, when cut into, stained the fingers with a sooty matter. The pancreas had lost considerably in bulk, but it had gained in consistence. Beneath it was found the splenic vein of usual firmness, and of a deep claret hue in its inner membrane.

Dr. Riley removed the stomach, with the lower portion of the œsophagus, the duodenum, the small and the large intestines, in separate portions; the rectum was taken out attached to the uterus and ovaries. On cutting the duodenum, a bright yellow substance escaped, but in very small quantity: the divided end being carefully compressed by Mr. Kelson's fingers. The parts thus separated were placed in clean vessels, and committed to the charge of Mr. Herapath.

*Thorax.*—The diaphragm was entire, firm, and, in its muscular part, of a darker tint than ordinary. The pleuræ had undergone no visible change; but the left contained a few ounces of very dark red fluid. The viscera were extremely collapsed, and lay at the bottom of the cavity, from which, together with the smooth and shining appearance of the pleuræ, it was inferred that there had been no adhesions during life. The heart was so shrunk and flattened, that it had been accidentally cut across in the process of removing the œsophagus; its lining membrane had a reddish brown color, having evidently been dyed by a fluid of the same tint which was contained in its cavities.

The head was removed by a section between the third and fourth cervical vertebræ, in case it might be required for identifying the individual by the teeth. The hair was of moderate length, brown and gray. The scalp peeled off very readily, as well as all the soft parts of the face, being converted into a thick saponaceous matter. The same adipocirous condition existed in the external parts of the neck. The soft parts of the fauces, and the pharynx with its neighboring tissues, were greatly decomposed, being soft, in some parts semi-fluid, and of a dirty ash color. The vertebræ were held together by their ligaments, pretty firmly, but the temporo-maxillary articulation was very loose."—"The extremities were white, firm, and rather plump. This condition resulted, apparently, from the partial conversion of the skin and subcutaneous cellular tissue into adipocire.* On cutting out a portion from the anterior surface of the thigh, I found the muscles beneath, claret-colored, fibrous, and rather soft. The aponeuroses and tendons were strong and shining, and apparently quite unaltered. The capsular ligament of the knee-joint was smooth and moist, except on the posterior surface of the patella. This bone was bare on the anterior surface, the integuments in this part having been destroyed, a circumstance which I attributed to the absence of adipose tissue in this part, and the consequent deficiency of the elements for margaric and elaic acids. The hair on the labia pudendi had not fallen off. The nails of the hands were firm, but those on the toes were very loose; and the saponaceous matter at the soles of the feet seemed but imperfectly formed. The epidermis had, in this part, separated, and adhered to the corresponding part of the stockings. The adipocire on the thorax was very thin, and more dry and cheesy than on the limbs: the anterior abdominal parietes consisted of little else, if we except a thin reddish layer of muscle, and the peritoneum. The cartilages of the ribs were soft, but quite elastic: they were very readily detached from their sternal connexions. I removed the base of the heart, with portions of the great vessels. These were not only firm, but even more rigid than ordinary, and were chocolate-colored on their inner surface. I also cut through the root of the left lung, and brought away about a third, attached to the above mentioned parts. The pulmonary parenchyma had lost its cellular structure, and was very soft."

The stomach cut open along the lesser curvature, by Mr. Herapath, presented a very striking appearance. "A thick and bright yellow coating, like paint, lay on the mucous membrane, particularly over the pyloric third: but it extended, more or less, with some small interjections of unstained membrane, to within two or three inches of the great cul-de-sac. A portion of this yellow matter was dried, then mixed with a little charcoal and carbonate of soda, and put into a reducing tube, and heated. A metallic silvery-looking crust, characteristic of arsenic, soon made its appearance in the upper part of the tube. Mr. Herapath, by heating this crust, in contact with atmospheric air, converted it into crystals of arsenious acid, which yielded the usual precipitates to ammoniacal nitrate of silver, ammoniacal sulphate of copper, and sulphuretted hydrogen." This series of tests was repeated five times. Mr. H. subsequently treated some mixed yellow-tinged matter, washed from the stomach, amounting to seventeen grains, in the following manner: thirteen grains were boiled in nitro-muriatic acid, which decomposed the animal matter, dissolved the phosphates and the arsenic, and converted the sulphur into sulphuric acid. Ammonia having been added in sufficient quantity to supersaturate this acid, the mixture was acidulated with acetic acid, and filtered. A stream of sulphuretted hydrogen, passed into it, precipitated four grains of sulphuret of arsenic. Mr. H. stated in his evidence, that the remaining four grains, out of the seventeen grains of mixed substance, would have yielded another grain of orpiment.

---

* On examining the specimen which I brought away with me more minutely than was possible in the church-yard, I found that the skin had undergone very little change beyond that of condensation, excepting also, that it was greasy to the touch; and that the cellular tissue beneath, though approaching in character to adipocire, was by no means so saponaceous as the substance on the face.

*Appearances of the stomach and intestines after ablution.—Mucus surface.*—In the cardiac extremity the color was dull red, or chocolate, diffused and uniform. There was no appearance either of distinct, injected vessels, or of red points. The texture was of the usual degree of firmness, neither softened nor indurated; and exhibiting nothing like hypertrophy and thickness on the one hand, or tenuity on the other; it was not in the least corrugated. The middle portion presented several indelible yellow stains, of indeterminate form; but in the pyloric third there were two remarkable patches, one about the size of the cut surface of a pigeon's egg, divided in its longitudinal diameter; the other somewhat smaller. Both of these spots had a well-defined margin, and were equally visible on the serous surface. The mucous surface, examined carefully through a lens, was observed to be perfectly entire, so that the very tissue was infiltrated with the coloring matter. Here and there small spots on the disc appeared rough to the naked eye, and simulated abrasion; but, on more minute inspection, this appearance was found to result from the membrane being, in those places, more particularly loaded with the matter alluded to. The same remark applies equally to all the other yellow patches on the villous coat, both of the stomach and intestines. In those portions of the pyloric half of the stomach, which were free from the pigment, there was a dark gray appearance, almost approaching to black; but no inequality of surface, or interruption of continuity, could be discovered. Elevations from hypertrophied glands, or from extravasations, were anxiously looked for, but without effect. The lining of the pylorous was of a light gray or ashy color, and had nothing else at all worthy of notice. The same may be said of the first two inches of the duodenum; but lower down this bowel presented a dark stain, similar to that in the stomach, above described. In the portion that lies in contact with the pancreas, there was a large yellow stain, the size of a dollar, across which an incision had been made in removing the part. About the commencement of the jejunum was another, the size of a shilling. The valvulæ conniventes were perfectly distinct, and in this spot infiltrated with the yellow substance; notwithstanding which they floated freely in liquid. In this intestine, the villous membrane was of a faint red, but became quite pale in the ilium, which offered nothing remarkable.

In each of the parts described, the mucous membrane was sufficiently tenacious to be lifted by the forceps, in as large flakes us usual.

In the cœcum and colon, an ash-gray color was noticed, differing more in degree, than in kind, from the dark stain in the pyloric portion of the stomach. The tissue was smooth, of its usual tenacity, presented no enlarged follicles of glands, was free from ulceration, and sufficiently transparent to render the submucous cellular membrane perceptible. The appearance of the latter tissue was peculiar; as seen through the mucous membrane, it gave me the idea of albumen coagulated in lines that intersected each other, and leaving distinct areolæ between them; the lines were of a dull pinkish color. The iliocœcal valve was, if any thing, somewhat more developed than usual. The hue of the villous coat of the rectum approached more to a chocolate or slate color, and was mottled with white spots of a circular form, depressed below the level of the surrounding membrane. Whether these depressions were owing to a loss of substance, or to the elevation of the rest of the tissue, was not easy to be determined; certainly they did not present the defined margins common to ulcers in this part. In my examination of the gray color of the colon, I noticed that the stain was communicated to the white board on which the part was lying, and was very similar to that of soot diffused in water.

*Serous surface.*—The most remarkable appearance on the outer surface of the stomach, if we except the yellow patches already mentioned, was a black diffused stain, of irregular form, over the superior and anterior surface of the pyloric half, or that portion of the viscus which usually lies immediately under the left lobe of the liver. It corresponded, in its deepest part, with the dark stain in the anterior. The color was deepest at the circumference ; and the general aspect was as if the viscus had lain in contact with some coloring liquid. The dark hue of the mucous lining of the duodenum, was likewise found to correspond with a similar coloration of the serous coat. In the part which was situated under the liver, I fancied that I could detect a similar correspondence between the color of the serous covering of the colon, and that of its internal membrane, particularly in the ascending portion. The mesentery was firm and healthy, and matted in a few of its convolutions. Some opaque white patches, however, were noticed, which, probably originated in enlarged or otherwise altered glands. It presented one or two slight yellow stains, which must necessarily have been acquired by imbibition. I noticed a similar appearance on the serous covering of the ilium, but the coloring matter had not transuded to the villous coat. In the meso-colon, also, there was one very bright yellow spot of circular form.

The appendices epiploicæ were perfectly distinct, but shrunken, hard, and somewhat cheesy.

The odor of all the parts above described, was *sui generis*, removed equally from their smell when examined in a fresh body, and from that of putrefaction. It was remarkably persistent ; I never had more trouble in getting my hands and clothes free from it ; and the same observation was made by others.

The uterus and ovaries were very small ; the latter barely distinguishable. The mucous lining of the vagina, and the os tincæ, were perfectly natural.

*Contents of the cranium, &c.*—I examined the head in the presence of Dr. Dick and Mr. Kelson, at the institution, on the morning of the 27th instant. It had lain in a macerating tub since the day of exhumation. The soft parts were much in the same state as before described, excepting that some of the tissues at the base of the skull were somewhat more decomposed. The greater part of the scalp, and coverings of the face, had been removed in the church-yard ; but what was left was completely saponaceous. The orbits presented no trace of eyes, they contained nothing but adipocire. There was no vestige of muscular aponeurosis in the temporal fossæ, and the pericranium was entirely gone. The sutures were easily recognised, but not less filled up than is usual at the age of the individual. The contents of the cranium were found to occupy not more than three-fifths of the cavity. The vacant space was in the back part. The dura-mater was in this situation, however, quite entire, and laterally also ; but in the region corresponding to the anterior lobes, it was found wanting, and here the cerebral matter was semi-fluid and putrilaginous. On removing, with scissors, the remains of the dura-mater, it was noticed to be quite as resistent as usual, and was smooth and shining on its inner surface. The lining membrane of the lateral sinuses had a dark red tint. The brain thus exposed was in the form of a globular mass, distinguishable into hemispheres, but barely into lobes. The pia-mater was clearly recognisable, and was traversed by one or two bright red veins. Its texture was so tender that I could scarcely separate it from the mass which it invested; but in my attempts to do so, the tomenta cerebri were very perceptible. The appearance of the cerebral mass was not unlike that of Stilton cheese, and its consistence extremely soft; but a section demonstrated the white and gray matter with perfect distinctness. The consistence prevented me from making any observation on the other parts. The tentorium cerebelli was quite firm, but beneath it was a pultaceous mass, in which it was vain to search for layers of cerebellum, tuber annulare, or any other nervous organ belonging to this region.

In separating the atlas from the occipital condyles, I found great resistance from the surrounding ligaments."

After offering some interesting observations on the singular state of preservation of the body, and the causes which may have contributed to this, with some comments on the appearances of the alimentary canal, Dr. Symonds proceeds to the consideration of the medico-legal inquiries arising out of the above investigation.

The first was "whether any thing had been found to indicate the causes of death?" Dr. S. thinks "that the pathological appearances afforded nothing but negative evidence, and that the discovery of the poisonous substance itself was by far the most important fact, with reference to the judicial question. The only question in this stage of the enquiry, was, how far the presence of this substance might be presumed to have been the cause of dissolution. Was it sufficient, *per se*, in the absence of proof of any other deleterious agent, to account for death? Had it been introduced during the life of the individual? The answer to the latter question could not but be affirmative, as the parieties of the trunk were entire; or if it were possible to conjecture that any one might have been ingenious and diabolical enough to have injected the arsenic by an elastic tube, passed along the mouth and gullet into the stomach, in order, at so remote a time, to support an imputation of poisoning, we still should find it difficult to explain how the matter could have reached the jejunum. The deleterious properties of the substance being admitted, was the quantity sufficient to occasion death? Had it been necessary to have answered this question, with reference to the quantity of arsenic which Mr. Herapath actually produced, the reply would have been all but affirmative. The substance was orpiment; supposing its base to have been arsenious acid, which was converted into a sulphuret, by the action of sulphureted hydrogen in the body, in accordance with actual observations of this kind by Christison, the discovery of five grains was nearly conclusive; or supposing that it had been given in the form of orpiment, as sold in the shops, the answer would have been the same, since the latter preparation contains more than nine-tenths of arsenious acid. But there was no necessity for limiting the question to so small a quantity, because much was wasted in the tests, and in the liquids employed in the ablution of the parts; while a considerable quantity remained incorporated with the tissues. It was thought probable by many, as well as by myself, that there must have been as much as a drachm altogether in the stomach and the intestines. When pressed in my examination to state what I believed to have been the smallest quantity, I said that I felt persuaded that there could not have been *less* than half a drachm. The substance and the quantity, then, being adequate to a fatal effect, did it actually produce this result? In the absence of any proof whatever, that any other fatal cause was in operation, there could be but one answer to this question. Nor do I think that the case would have been different, even had we discovered the traces of organic disease in some vital organ; since the experience of every practitioner must have taught him, that the fatal results of such lesions have no determinate time, while the period of action for a poison is far more definite, and consequently, where both are discovered, it is reasonable to ascribe death to the latter, though there is a remote possibility that the effect of the former might have accidentally taken place before the poison had had time to complete its work. But there is scarcely any, even the most acknowledged mortal agent, which is not liable to the exception of such a possibility. On finding the appearance of intense enteritis, the inference that this disease was the cause of death might be questioned, if nothing was known of the history during life, on the possibility that some sudden external cause, such as a concussion, or a stroke of lightning, might have cut off the patient. Such refinements are, however, insufficient to divert the belief from the more obvious conclusion; the highest degree of probability in such cases must amount practically to absolute certainty.

That arsenic, then, had occasioned the death of the deceased, was the opinion formed by the medical witnesses upon the results of their examination of the body. How far we were supported by the account of the occurrences during the last hour of the individual's life, will appear, in part, from the following abstract of the evidence of two persons who attended upon the deceased, and partly from the confession of the culprit, made after her condemnation. Mary Ann Allen, the principal witness for the prosecution, stated, among many other circumstances, the greater part of which belonged to the proof of administration on the part of Mrs. Burdock, and which, therefore, need not be inserted here,—that she saw the deceased, for the first time, on the evening (Thursday) before her death; that she was warned by Mrs. B. never "to eat any thing after Mrs. Smith;" that the deceased was in weak health, and kept her bed, but was not very ill;* that on the following morning, the deceased expressed herself as being much better, and hoped to go down to her parlor on the ensuing Sunday; that the deceased attributed her indisposition to a cold taken by walking out on a windy day; and that nothing remarkable happened till the evening, when Mrs. Smith appeared more poorly than previously, and declined taking some gruel, because her mouth was sore. In the course of the evening some gruel was administered, witness having previously seen a yellow powder mixed with it in another room, which powder she was told was medicinal; but she stated that,

* At the coroner's inquest, this witness deposed that the deceased labored under diarrhœa, and that the evacuations were very dark-colored.

before this addition, the gruel had a reddish color. About half the quantity in the basin was drunk, and shortly afterwards Mrs. Smith appeared in great pain, "rolling about the bed in great agony;" she did not vomit, but spat a bloody matter: after a time she became quiet, and seemed asleep. No other symptoms were mentioned by this witness, except that, after the interval of quiet, the deceased raised her head convulsively, struck it against the head-board and expired. Witness had no means of computing time, but supposed that about two hours had elapsed between the taking of the gruel and the death. There is reason, however, to believe that, upon this point she was mistaken, and that the time was much longer. Another girl, Charlotte Thomas, who had been employed in a similar capacity to that of Allen, for nine days previously to the engagement of the latter, and who was the principal witness for the defence, declared that the deceased had a very sore mouth;* was in the habit of using a gargle, and spat a great deal of blood. The witness left Mrs. Smith in consequence of her own health being seriously deranged; and it is deserving of notice, that among her ailments was a sore mouth.

I need scarcely remark, that very little exactness could be expected in the detail of symptoms observed, fourteen months previously, by Allen, a person not more than fourteen years of age at the time, and unaccustomed to wait upon the sick. I heard her deliver her evidence both at the inquest and the trial, and gathered from it that Mrs. Smith, previously to taking the gruel, as she called it, (because it had been so designated by the culprit) was very unwell, but became much worse soon after. Supposing her account of the time to have been correct, and that the arsenic had been administered for the first time in the liquid mentioned, the fatal event must have taken place sooner than in any case actually upon record. Dr. Christison mentions three hours, as the shortest period that he has met with; but he alludes to a case tried at Warwick, mentioned by Mr. Evans, of Lewes, in which death was said to have occurred in two hours. In the present instance, as I have already intimated, there was reason to think that the time had been miscalculated; but even if double the period had elapsed, it was extremely brief, and the case must have been classed among those in which death is brought about by the remote action of the arsenic upon the contractility of the heart. The absence of vomiting would tend to corroborate this opinion, and, perhaps, also, the want of any signs of irritation in the *post-mortem* appearances. It was, however, quite open to conjecture, that a dose of the poison might have been given previously; and my professional brethren, and myself, were strongly inclined to this opinion; though, if evidence had been brought to show that this could not have been the case, it would not have altered our conviction respecting the cause of death; for, without adverting to the fact that there is an individuality in every case, which may render it an exception to all general rules, there was nothing unreasonable in believing that an irritant poison would operate more rapidly upon a person already enfeebled, especially when it was known that the dose was considerable. In the defence, no attempt was made by the prisoner's counsel to elicit any doubt upon this subject.

---

* At the inquest she spoke of "holes" in the mouth and gums, and great swelling of the face.

The account given of the previous condition of the deceased, very naturally suggested the idea that she had been under the influence of mercury, and it was not uncharitable to conjecture that the individual who had administered the arsenic, had previously made an attempt with corrosive sublimate. It was remarkable that the girl Thomas left with a sore mouth, and that Allen was particularly enjoined not to taste any thing after Mrs. Smith, an injunction which probably had either not been given to, or had not been followed by Thomas. The opinion upon this subject, formed by most of the professional men who heard the evidence, is further supported by a fact which I have received from credible authority, namely, that Mrs. Burdock had frequently purchased at a chemist's shop, small quantities of corrosive sublimate, under the pretext of using it medicinally. The diarrhœa, testified by both the witnesses alluded to, adds weight to the suspicion; and not the less so, if the *post-mortem* appearances in the large intestines indicated chronic inflammation.

"After her conviction, Mrs. Burdock, in a somewhat scanty confession, or declaration, to a woman employed to attend her, stated that arsenic had been given to the deceased on the day before her death, and a larger quantity on the following evening, as the former dose appeared insufficient. The only discrepance between her statement and that of Allen, was, that the liquid was thickened milk, and not gruel. She made no remarks, that we have heard of, upon the symptoms of her victim. By this declaration, the peculiarity as to the shortness of time occupied in the action of the poison, is done away with; but it is somewhat remarkable that nothing should have appeared upon evidence tending to show that Mrs. Smith labored under irritation from the effects of the first dose, (unless we admit that the diarrhœa was more recent than is intimated by other testimony,) or that she was worse than usual until the following evening. Some may, perhaps, conjecture that vomiting might have partially removed the substance, though there is no evidence of such an occurrence. I should be more disposed to think that there was a deficiency of observation, or of memory, upon the subject, which is quite conceivable with reference to so young an attendant as Allen, and perfectly compatible with the value of her testimony upon other facts, of which she was more capable of taking cognisance, and preserving the recollection.

"I shall now make one or two remarks upon the poison which was used in this case. It has been already stated, that the substance detected in the body was the yellow sulphuret; but it did not follow that this was the identical preparation that had been administered, because arsenious acid may be converted into the sulphuret in the dead body.* It appeared in evidence, however, that the substance purchased and used in the case was called yellow arsenic by the chemist who sold it. Having procured a specimen of this preparation, in order to try its activity upon animals, I found that it did not correspond in color either to common orpiment, or to the spots in the stomach. It inclined more to red than yellow, and in this respect resembled realgar, but was still not so red as the latter compound. When mixed with gruel

* Dr. Christison was the first to point out this fact. In the "Exhumations Juridiques," (t. 1, p. 221) already cited more than once, there is a case in which yellow spots in the cardiac extremity of the stomach were observed, and grains of white arsenic in the ilium.

it gave a rich golden tint; with milk it inclined somewhat more to yellow; and with water the color was a dull orange. It consisted of a fine powder, not easily miscible with liquid, and a great number of little grains and lumps. Some of the latter, when freed from the powder, were found to be orpiment, and others white arsenic. I subsequently examined the contents of the chemist's jar, and found in it several large lumps, some of which were cakes of orpiment, but most of them were of an irregular crystalline appearance, consisting of arsenious acid, streaked with yellow lines. I have not been able to ascertain the precise mode of manufacturing orpiment in this country at the present time. On the Continent it is made by subliming sulphur and arsenious acid together; and the analysis of the substance given by M. Guibourt, is ninety-four parts of arsenious acid to six of the sulphuret.* In Dr. Mead's time,† 'yellow arsenic was prepared by subliming white arsenic with the addition of a tenth part of sulphur. 'The red,' he adds, 'differs from the yellow only in this, that a greater quantity of sulphur is added, together with a particular kind of a red cobalt called kupfer nickel.' It is not probable that any important difference has been made, if any, from the above proportions. The druggist informed me that the substance in question was part of an old stock that he had taken from some other person in the trade; and I conjectured that it had probably been a sort of refuse left at the bottom of the alembic, after the sublimation of a certain quantity in a pure state, to be used as a pigment; and that this residuum had been broken up, and kept for coarse purposes, such, for instance, as that of poisoning vermin. Mr. Herapath analysed the preparation, and found it very variable, from the irregular intermixture of the grains of arsenious acid and orpiment. His general result was, that one hundred parts of the red powder contained seventy-nine of arsenious acid, and twenty-one of realgar. He found that a stream of sulphuretted hydrogen gave it a bright yellow color.

"'The comparative inertness of the native or pure sulphurets is only what might be expected from their composition. When they act deleteriously, it is, probably, not till the metallic arsenic has derived oxygen from the organs or their contents, or from the liquid in which they may have been administered: we owe to M. Courdemanche the discovery of the important fact, that sulphuret of arsenic and water may, by the decomposition of the latter, yield arsenious acid and sulphuretted hydrogen.‡ With the yellow arsenic of commerce, M. Renault killed a small dog in five hours: the dose was four grains. A larger dog was killed in nine hours, with three grains. When applied to a wound, in quantities of a drachm, eighteen grains, and eight grains, it caused death in from fifteen to eighteen hours.§ No precise experiments, that I am aware of, have been performed with *artificial* realgar. Orfila states, in general terms, that a few grains are fatal '*au bout d'un temps variable*;' and relates the following case, taken from the *Ephemer. Nat. Cur.*: '*Une femme mourut dans l'espace de quelques heures, apres avoir eprouve des tranchees violentes, pour avoir mange des choux, au quels on avait mele une certaine quantite de cette substance.*' It is difficult to understand how it should be so

---

* Dict. de Med. Pratiq. et de Chirurg. Art. Arsenic. †Mead on Poisons, p. 218. 1745.

‡Orfila—Lecons, de Med. Leg. t. 3, p. 177. §Orfila—Toxicol. Gen. t. 1, p. 450.

active, if the analysis given by M. Guibourt, on the authority of M. Laugier, be correct, viz. that only 0,015 of its weight consist of arsenious acid."—*Ib.*

16. *On the Climate of Madeira, and its true value to the Consumptive.*—By J. M. Calvert, M. D. The situation of the island in the midst of a very large ocean, the latitude, the position of the town of Funchal, &c. all tend to produce great uniformity of temperature and great moisture, by the common principles of meteorology. The moisture is, of course, little seen in the town itself, being kept in such perfect solution by a uniform temperature, but it is generally visible, in the shape of a fog, on the side of the mountain above, whilst its existence is certain below, not merely from theoretical reasoning, but from the observations which have been frequently made there: particularly from the full and scientific tables of Dr. Mason. I avoid, however, all theoretical reasoning, both regarding the climate and also disease, at present: wishing merely to give a few practical hints as shortly as possible. I therefore only add that the feelings of all persons when they first go, both sick and healthy, those benefitted and those not, fully bear out the meteorological conclusions. Hence the climate is extremely useful to those patients who suffer from a dry irritable state of the mucous membranes, whether local or general; and in order to avoid a detail of symptoms, I may express them all under the *strictum* of the ancients: they are well detailed in Dr. Clark's work on Climate; and he recommends Devonshire, Guernsey, Jersey, &c. for them, and it is for these symptoms that I recommend Madeira; a winter here being, in many respects, a summer in those places. On the other hand, I am very anxious to express my strong conviction that, in the opposite class of symptoms, the *laxum* of the ancients, particularly if there are present symptoms of increased mucous and still more of purulent secretion, hemoptysis, a tendency to relaxed, or what is called a slippery state of the bowels, if moist and emolient treatment have disagreed before, that in all these cases the climate will do harm in proportion to the good in the contrary: it will, indeed, often, in cases of general constitutional relaxation, do good at first, by removing some local irritation brought on by disease —as a dry cough produced by tubercles—but it will subsequently increase these by increasing the constitutional disorder which was connected with their formation. Such patients, therefore, must change their climate just as they would change their medicine, with change of symptoms.

All that Dr. Clark has said about the impropriety of sending patients in the advanced stage of disease is quite true; and I am only anxious to follow out his remarks by saying, that not only must the symptoms be slight, but they must also be of the peculiar character I have mentioned. The vague idea that Madeira has the finest climate in the world, and therefore is applicable to all cases of lung disease, is just as absurd as a similar notion about any valuable medicine; and the idea has hastened the death of many who might have long lived by means of a drier air, slight tonics, &c. It is important also to recollect that this drier air is not to be found in the island: the higher grounds are very generally covered with fogs in winter, and the few houses there are in summer occupied by the owners; besides that, the air does not become drier as we ascend, but only cooler; at least it remains equally near the point of saturation, which is a very important distinction. Hence the invalid must look to the town, and to it only, as his residence; and consequently eight out of ten must leave the island in summer, or their symptoms will be aggravated, as consumptive symptoms so often are in very hot weather, and as has happened to a friend of mine who remained in the island this summer. The town of Funchal (and it is the only one) was full last winter, and should the cholera send more persons there now, it is probable that great inconvenience will be felt by many. The voyage there, and still more the difficulty of getting home, is a much more serious thing to an invalid than those who have not shared in their sufferings imagine: many certain, and more contingent evils, both moral and physical, beset the patient from the moment he leaves home till he reaches it again; and these ought, as far as possible, to be explained to him. If tubercles, in however slight a degree, are known to exist, let him be told what is the utmost climate can do for him, or his distress will be very great, when, far from home, all the Utopian delusion connected with the word Madeira is torn from him. Here, however, I must strongly recommend the perusal of the first and second chapter of Dr. Clark's work, in which he insists on the care necessary in diet, clothing, medicine, &c. &c. for those who go abroad; the negligent way in which many patients are sent off, with no other advice but to give up all medicine, and trust to that ill-understood thing "climate," is, I know, the cause of irretrievable mischief. The patient very naturally persuades himself that climate will compensate for every imprudence, instead of being, at best, but the most favorable opportunity for care and good treatment. A winter in Madeira, is, to all practical purposes, but a summer in Devonshire or Jersey; whilst there are many peculiarities in it which make care in all the common habits of life still more necessary. Recommending, as I do very strongly, Dr. Clark's work, to all who travel for health, he will, I hope, allow me to say that I think he has over-colored Madeira, and I must suggest two hints to his readers; that Madeira is, with a few exceptions, fit for a winter residence only, and that it does not combine the advantages of two kinds of climate, but must strictly be classed with the warm and moist; as Pau, Devonshire, &c.

I have intentionally dwelt more on the evils than good of Madeira, in this short letter; my object being to prevent those who are going to the dry bracing air of Nice, &c. from ignorantly turning round with the idea of finding it at Madeira; this being, in fact, just the contrary. My difficulty in writing has been not to dilate, and I would gladly at this moment state the advantages of the island: this I may probably hereafter do, in conjunction with one or two friends; whilst I must for the present be content to say that the good, in well-selected cases, is proportionate to the evil in the contrary, and to shew that I am impartial, I may add that it is not improbable I may, before the winter is over, go back to the island myself.—*London Med. Gaz.* 24th Oct. 1835.—*Ib.*

17. *Medical Instruction in Paris.*—M. Guizot, the minister of public instruction in France, in his report to the king relative to the creation of a chair of pathological anatomy in the Medical School of

Paris, states. "that instruction in the Faculty of Medicine, of Paris, though enlarged by the successive creation of different chairs, is still, in some respects, not on a level with the existing state of medical science." Louis Phillippe seems to have coincided in this sentiment, for he has issued an ordinance for the creation of a new chair, to be supported by the legacy of M. Dupuytren. Nevertheless the faculty of medicine of Paris, prior to this ordinance, comprised sixteen chairs, with twenty-three professors, and thirty-six aggregees. In this country, so far as we know, there is no medical school in which there are more than seven chairs; and some have only three! Is it because medical science is at a lower ebb here than in France, or because our schools are not on a level with the existing state of the science? We feel strongly tempted to dilate upon this subject, but are compelled, by want of space, to forego, at present, the indulgence of our inclination. We leave it, therefore, for the sages who regulate our medical institutions to inquire into the matter; but, as little satisfaction to the profession is to be expected from this source, we shall take an early opportunity of offering our own solution of the questions.—*Ib.*

18. *Cases illustrative of the effects of Iodine, used externally.*—By A. Campbell. The value of Iodine, as a remedy in discussing glandular tumors, be they of a peculiar nature as Goitre, or depending on a scrophulous habit of body, is firmly enough established among the European portion of the medical world. The fact is sufficient for all purposes of relieving a portion of human misery. As to the mode of its action, it is now, and probably will remain, as inexplicable as most other physiological phenomena. I shall not bother myself, you, or your readers, with mystifying attempts at satisfactorily accounting for the curative powers of the drug, through the means of an explained *modus operandi*, but note the few cases of my small experience, to be added to those of other people.

*Case 1st.*—May 10th. Narayani, a Hindoo female, aged 25, of good caste, very fine complexion, large and prominent jet black eyes, the mother of one child, and marked with the pits of small pox, has swelling of the glands on both sides of the neck, with ulceration of those on the left side, and copious discharge of white curdy pus. Six months ago, after her recovery from the small-pox, and two months after the birth of her first and only child, the enlargement of the glands commenced. The right swelling went on slowly, and without pain; the left one increased rapidly to suffocation, and the ulcerated surface now is three inches long and two inches in breadth: the glands under it hard, and much enlarged; general health excellent: slight redness of conjunctiva of both eyes, with weakness of sight. Pledgets with ointment of hydriodate of potash to be applied to both sides of the neck, with poultices over them. To take ten grains of the compound rhubarb pill every night.

May 15th. The entire gland softer and much reduced in size; the ulcerated one became very painful the day after the application of the ointment, when, the ointment was reduced in strength. Now the discharge is diminished; is not so scrophulous-looking, and the ulcerated surface is clean, and the edges of it are beginning to smooth down: body of gland softer and smaller. Continue remedies, omitting poultices.

22d. The ulcer has gone on healing and the glands diminishing since last report; swelling of entire gland gone; ulcerated surface looking well, but rather indolent. To be dressed with the ointment—of original strength. The pills every second night.

30th. Ulcerated surface skinned over and much smoother than cicatrices of the like generally are;—swelling of the gland gone, and the lady is well pleased at the change from loathsome sores to healthy cuticle.

*Case 2d.* A Nipalese soldier, aged 24, who has been under my care for the last two months for the cure of chronic ulcers on the right leg, is still afflicted with some of the ulcers round the external ancle. He is not, apparently, of a scrophulous habit. I have tried adhesive strap, bandaging, and the lunar caustic treatment, with success in the ulcers on the tibia and calf of the leg, but not in those lowest down. Will try the iodine.

June 4th. Dress the ulcers with the hydriodate of potash ointment, and apply cotton roller up to the ulcer.

June 12th. The ulcers are much improved in appearance: firm granulations are springing up, and the edges all smoothing upwards. Continue.

24th. No retrogradation: healing has gone on regularly, and he is now quite well.

*Case 3d.* This is an anomalous sort of affection. A Mussulman lad, about 18, had been for many months affected with fetid small ulcers, about the edges and insides of the nostril. I had used various washes and unguents; under the use of a strong solution of the sulphate of quina and the citron ointment; the sores healed several times, but as often returned. I smeared them daily with the hydr: of pot: ointment for a week; when they perfectly healed, and have not, as yet, (three months) returned.

*Case 4th.* Our fair countrywomen may take a hint from, and benefit by, the result of this case, despite of Rowland and his incomparable Kalydor. A young and pretty nautch girl was my patient. She had somehow or other (may be in an affray with a rival or rude lover) got her forehead bruised and scratched: the wounded part had soon healed, but a spot the size of a sixpence remained *quite black.* She was fair to look upon for a native of our burning plains, and vain as fair. The black spot was the temporary plague of her life;—she had used innumerable recipes, prescribed by natives, but without effect; when, at last, she beseeched of me to remove the deformity. I gave her some of the hyd: of pot: ointment, to be rubbed on the spot, and to my surprise, and her great joy, at the end of a week, there was not a vestige of the discoloration. I have used the ointment three times since in cutaneous discolorations, with the same success.

The next and last case is not quite germain to its predecessor, but its result, if confirmed by further trials, would be of inestimable benefit in India, where horses are so great a comfort, and where they are so subject to disease peculiar to the season of the year.

I have an Arab horse (most people have) who, for the three last rainy seasons, has had spots of *bursauttee* on the inside of the thighs and fore-legs: they disappear in November and return in July. This year, as usual, they appeared. About the middle of last month (August) I commenced applying a wash, made of equal parts of the tincture of iodine and water, morning and evening, to the sores, and in twelve days from the first application, they were perfectly healed, and now their sides are nearly covered with hair. I have used nitric acid and sulphate of copper washes, but not with half the success of this iodine one. The fact may be useful to some readers whose horses are affected with this troublesome disease.

In the two first cases noted, and in a few others similarly treated since, it was necessary to watch the effect of the ointment on the ulcer. When it gives much pain, and appears to stimulate the ulcer too much, it is neceesary to add lard or simple cerate to it. I make the hydriodate of potash ointment with two drachms of the hydriodate, to one ounce of simple cerate as the standard: to be made stronger or weaker as the effect on the ulcer may seem to indicate.—*India Medical Journal of Science.*

19. *Bleeding in the cold stage of Intermittent Fever.*—The novelty of this practice, and the dangerous consequences supposed to result from it, have deterred many from pursuing this mode of treatment. It is but natural that we should receive with distrust and adopt with caution, a doctrine, which seems so totally at variance with all our preconceived notions of the nature and treatment of this disease. The employment of V. S. is one of the newest lights, which has dawned on us, and is, I believe, a coruscation from the luminous and inventive imagination of Dr. Mackintosh of Edinburgh, assisted by an unwearied assiduity, and perseverance, in every object connected with the profession; to this gentleman we are indebted for a safe and most useful prophylactic, by the judicious administration of which, this most troublesome disease it not only speedily cured, but the chronic visceral derangements, so frequently the sequelæ of its invasion, are in a great measure, if not wholly obviated.

The practice is, I believe, far from being universal in this country, and I am aware that medical men in general, are averse to it, particularly among the natives; I confess that, at first, I felt some difficulty in overcoming my own prejudices, and with a wholesome dread of fatal syncope, debility, natural want of stamina, I, with a trembling hand, resolved to try its effects on an emaciated *kidmutgar*, who was ill for six days previously. Having directed him to come to me when his cold fit was about to commence, I took from his arm six ounces of blood, when he became faint, and continued in that state for nearly half an hour; giving him, every now and then, a little *ammonia*, he soon called and walked home, without the assistance of two friends who had accompanied him to my house. The fever was of the quotidian type; I gave *two* grains of quinine to take the following morning. He has never since had a return of the symptoms, and enjoys his usual health. My next patient was a prisoner in the jail, a stout, robust fellow, attacked with fever of the tertian type, had one paroxysm, the hot fit extremely severe, violent head-ache, skin hot and dry, the intermission perfect—has had no medicine; ordered him to be bled when the next cold fit had fully commenced. The result was as I anticipated, the paroxysm was instantly arrested, the hot and sweating stages scarcely observable, and no return of fever; ordering him some purgative medicine, he was discharged—four days subsequently, he got intoxicated, which brought on a return of his former symptoms; he was, by his own particular request, treated as before, and with the same agreeable result; the quantity of blood taken in the first instance amounted to twelve ounces, in the second to ten ounces. A servant of my own was the next patient on whom I tried its effects; his fever was of the quotidian form; I bled him when the cold stage had fairly set in, after which he had no regular return of fever, except that for two days after, a slight disposition to recurrence of the symptoms manifested itself, which yielded readily to small doses of quinine: except a purgative, he took no other medicine, and has since continued well. It would be useless to enumerate the many examples I have had of the efficacy of this plan. The two last months have afforded me many opportunities of putting it to the test of experience, I can therefore with confidence recommend V. S. as an invaluable remedy in this disease.

There are, however, some cases where it may be injudicious and even dangerous to adopt it, where in old ex-sanguineous subjects great debilty and a complication of other diseases co-exist, but these are, I apprehend, few. In a case which I had lately under my care, in which the propriety of V. S. appeared questionable, I witnessed the best effects from the topical application of leeches.

An old servant, laboring under chronic enlargement of the liver, and an enormous spleen, has had tertian fever for the last sixteen months, for which quinine and various remedies have been taken in vain, applied to me lately; on examination I found that he suffered little from the diseased liver, but complained a good deal of the spleen, which became extremely painful during the fever, particularly in the cold stage. I directed the application of six leeches for three successive periods, with a calomel purge: from this mode of treatment he derived the greatest benefit; pain and tenderness of the spleen much diminished, and a postponement or irregularity of the paroxysms, with a sensible decrease in severity—the subsequent exhibition of quinine, which before proved useless, now manifested its usual powers as a febrifuge, and the poor man continues free from fever, his other complaints nearly in statu quo.

In this instance, it is, I think, clear, that fever was kept up partly from the influence of habit, but in a much greater degree from the diseased spleen being unable with impunity to bear the increased influx of blood, which is directed in an especial manner, to this organ in intermittent fever.

I abstain from offering any observation as to the "quo modo" of its action, this being a subject which requires for its proper consideration, more time and talent than I can bestow. I should hope that some one more competent for the task would undertake it, content to add my humble efforts to that of others, in recording the advantages to be derived from the employment of a remedy at once simple and efficacious.

I cannot conclude without observing, that I feel myself indebted to Mr. Twining for having drawn my attention, and, I believe, that of the profession in this country to this subject—a circumstance which, in itself, entitles the practice to favorable consideration, coming forth, stamped with the authority of one whose zeal in scientific pursuits is always so conspicuous, and whose unbounded experience and sound judgment, so fully qualify him to form a correct estimate of its value.—*Ib.* T. C.

20. *On the stimulating effects of Cold Water.*—By Mr. Davidson. I have lately read an account of a recovery from the effects of lightning by the application of cold water, in one of the numbers of your Journal, and perhaps you will not object to the publication of the following circumstances, which may be relied on as perfectly free from exaggeration.

I was once called by my servants while at Asseergurh, to kill a large powerful snake that was seen on my farm-yard. After a really desperate fight I killed a dhaman six feet long, and, as I then thought, smashed its brains to atoms with a heavy solid bamboo. I took it up in triumph, and carried it to the front of my bungalow; as I wished to preserve it, and it had become very dirty, I poured a gharrah of water over its head, when to my great surprise, it immediately began running down the hill, and I had to kill it over again. I believed that the reanimation had been caused by the application of the cold water. Returning from Bombay in 1823, I passed through the cantonments at Jalna, then occupied by Madras troops. I called one morning on Dr. Alexander, who was attached to the horse artillery, and he told me that he had, in that morning's ride, rode down and killed a sickly wolf, with his hunting whip. I wished to see it, and was taken to the compound hedge, where the animal, to all appearance, lay dead. I said, "I'll make that wolf get up and look you in the face." Alexander laughed, and said that he had smashed its scull with the heavy brass end of his whip, and trailed it lifeless after him for a mile or two. I still persisted that I could re-animate it. At last he said, "Very well, try." Some cold water was procured, and on its being dashed over the scull, the animal got up, rested on its fore legs, and stared at us both with a most horrid glare! If Dr. Alexander be alive, he will probably remember his astonishment on the occasion.

In consequence of these and similar occurrences, I began to place great confidence in the power of cold water applied to the brain; and I will now relate, in my opinion, a very extraordinary circumstance, which shows that it was not without good cause. Soon after my return, one of my chupprassees solicited permission to be absent for a few days, for the purpose of visiting a large mela or fair, held in the middle of a dark and gloomy forest, 20 miles to the east of the fortress. Being fond of seeing sights, I determined to accompany him, and after having dispatched a tent, on the second day I rode to a village half way, and slept there during the night; next morning I reached the fair, which was held on an open spot in the centre of the forest. It was a festival in honor of one of those huge striding Hindu deities, who, from the peak of the Himalaya, placed one foot on a rock in the middle of the fair, and at another step stood on a mountain in Ceylon! There could be no doubt of the fact, as the impression of his foot was still visible on the rock; and it was visited once a year with great reverence, by 150,000 innocent 'Hindus.' Nothing occurred during the day, but in the night I was repeatedly awakened by a simultaneous and universal clapping of hands in honor of the deity before mentioned, which, in the stillness of the forest, had a very curious and startling effect.

I got up at day-break next morning, and, mounting my horse, traversed the fair in all directions, at one time mightily tickled by observing faqueers lying naked on the thorns of the Babul (most *care-*

*fully crushed*), and at another admiring the simplicity of the innumerable devotees who were crawling up and down the hill, to visit the sacred spot. On returning home I perceived, at a considerable distance, on the skirts of the fair, two large mobs. I cantered up to the nearest, and instead of seeing some outrageously pious Hindu Saint, observed a very stout and handsome young woman of about 20, lying apparently dead on the ground. I quickly dismounted, and found no signs of life, but warmth of body. There was no pulsation at the wrist, temporal artery, heart or ancle. On enquiry I ascertained that she had been lying two ghurrees in that state, having fallen down *suddenly* without previous notice or illness. Owing to the mob round her, the heat was perfectly suffocating, and I desired her husband, a grey-headed old man, to clear a large space round her for the sake of air, and I would try to cure her. This he eventually did, with the assistance of other men, but with great reluctance; as he said she had been so long dead, that he *must take her to be buried.* She was a Mussulman; I proceeded to examine the body more minutely, and all that I could discover was, that the attack was not epileptic, but might be from spasm.

I called out for cold water, but as none was procurable, I dispatched my groom, who brought my servants from my tent with a couple of large gharrahs, meant for my morning bathe, which had been cooling all night. The mob was again cleared, and sitting down, I dashed water on the woman's head two or three times, still there were no symptoms of returning life. I tried again and again, and at last one of the eye-lids twinkled. Delighted at this, I cleared the ground again, and uncovering her chest, I poured a whole gharrah over her body.

The effect was magical; she suddenly started up with a scream, wiped the water from her face, seized her dishevelled hair, twisting it behind her head, snatched her clothes, and looked round with a stare of astonishment. A loud shout of surprise rent the air, the miraculous cure of the feringee spread like wild-fire through the fair; accompanied by her sister and husband, I walked to my tent, surrounded by a dense crowd of admirers! After seating the party in my tent, I rode off to the other mob, where lay the body of a boy of ten or twelve years of age, surrounded by howling females. I knelt down and found his body also warm, but without pulse. I requested that the mob might be cleared, and offered to try my skill on him, and my request was backed by many who accompanied me from the first scene, but all in vain. I was obliged to leave the poor boy to his fate, and was returning to my tent, which I had scarcely reached, when I saw him carried past with his legs dangling in the air, to be, in my belief, buried alive.

I found my patient's pulse very languid, and therefore gave her a pint of old Madeira at a draught, which, 'nothing loth,' she swallowed, just to give a *filip* to the circulation! She slept soundly till sunset, when, without once thanking me, she walked out of the tent.

The fair lasted two or three days after this, during which time I never left my tent without being followed by a mob; and I am persuaded that if I had felt inclined, I might have set up for a prophet, without further capital, and procured a lakh of chelas in twenty-four hours. But you know that is not my vocation!

A year afterwards, when riding through one of the streets of Booraupoor, I overheard a conversation amongst some Mussulmans, who were disputing who were the most learned? the Moosulmans or Feringees? One vehemently backed the feringees, and swore "Koran ki Kusum that there were none so clever, for with his own eyes he had seen one of them raise a dead woman to life." "Jhooth bat," said another. I laughed; the Philo-feringee jumped up and roared out, "Soobhan Ullah, there's the sahib himself, ask him. Krors of people saw him do it."

Since that period, I have repeatedly stopped fits of epiplepsy in people found lying in the streets of different towns, by the same means.—*Ib.*

21. *Various effects of Iodine.*—By T. C. In former Nos. of your Journal are detailed cases of *Glossitis*, in which the usual antiphlogistic treatment were had recourse to with success. The following case being somewhat different as to symptoms and treatment may probably induce you to give it a place in your columns. It may be termed *Chronic Glossitis.* The subject of it was an old Mussulman writer, who stated that the enlargement was of six months' standing, had been gradually increasing, now protrudes beyond his lips, and totally obstructs the passage of solid food—in other respects he enjoys tolerable health. His disease commencing during his recovery from a severe attack of remittent fever, unaccompanied with decided pain, occasional slight lancinating twitches in the organ, being the only symptoms which arrested and directed his attention to a close examination of the part. The swelling presents a red shining appearance and a soft elastic feel, as if it contained air, uniform throughout. Fever subsided without medical treatment. It occurred to me, to scarify the part. as the usual mode of treatment adopted in similar cases, but the patient strongly objecting, I was satisfied with the application of four leeches, which bled freely, but without affording any relief. I then had recourse to blisters external to the swelling, and applied three successively, without the least perceptible advantage. Mercury or Iodine now seemed to me to offer the only prospect of success that remained, I therefore, with a hope that has seldom been disappointed, determined on applying iodine in the form of an ointment on the surface abraded by the blisters, in the proportion of ʒ ss. of iodine to ʒ ss. of Resin ointment, small portions to be rubbed in three times daily—in the course of ten days, decided amendment became visible, the tongue reduced to nearly its natural size, deglutition and respiration performed with perfect ease.

In many points of view the case is, I think, not undeserving of notice, being an interesting and novel termination of the previous fever, and a beautiful exemplification of the variety of modes which nature adopts, to expel from the system that morbific principle upon which diseases, fevers in particular, are, by many, supposed to depend.

It furnishes also another proof, if any now-a-days, be necessary, of the powerful, and I may add, specific effect of this medicine and its various preparations, in combatting many diseases, which, before its discovery, were considered beyond the power of the therapeutic art. We possess, in this medicine, an agent, which exhibits over the absorbent system in particular, an almost unlimited control; and I believe, there are few complaints in which it has not been tried, and in general with more or less success, and under judicious management, never with decidedly injurious effects.

Being partial to its administration, I use it with advantage in all glandular strumous swellings, dismennorhea, and goitre, and as yet never have had reason to regret its employment. For internal exhibition, I consider the hydriodate of potass the most eligible, either in the liquid or solid state, being equally powerful with the other forms, and less liable to induce inconveniences, which do sometimes follow the continued exhibition of iodine.

That distressing consequences sometimes arise from its abuse, I should be *sorry* to deny. This circumstance of itself being a proof of its activity, as a remedial agent, being in fact its most powerful recommendation, and not inferior in that respect to mercury and the preparations of it.

These remarks may be considered needless digressions, "vox et præterea nil," but I am induced to make them because of the growing feeling among many of the profession to underrate the value of iodine amply shown in our scientific journals, by clinical details of its malignant effects in some instances—and I know from experience, that a feeling, any thing but favorable to its employment, is daily gaining ground among those whom we are called upon in our professional capacity to attend—verbum sat.

Unqualified or exaggerated praise is as imperious as unsparing censure, and both tend equally to bring the most important discoveries into contempt—we should, therefore, when we discuss the merits of a question, medical or moral, take care that a good cause may not suffer from the injudicious employment of either the one or the other.—*Ib.*

22. *Account of the Burmese Practice of Midwifery.*—The following account of the practice of Midwifery among the Burmese will excite the astonishment of medical men more in Europe, than those in India, to whom the barbarous and cruel system is familiar. This statement is authenticated; and although the author is not willing to give his real signature to it, his accuracy, in regard to the circumstances he has brought forward, is not to be questioned. How loudly does the distressing description call for professional tuition in the obstetric art among the natives? and it strengthens the position we took up in our comments on the new Medical College, that a professor of Midwifery, who also would give lectures on female disease and those of children, should be added to the present number of professors appointed for that institution. When we make such suggestions as these we are reminded of the expense; but how can the monthly expense of a few hundred rupees be thought of for a moment, when by the expenditure of such a trifle thousands of lives may be saved, a variety of diseases prevented, and the number of deformed cripples which are now seen all over India be lessened.

*To the Editor of the India Journal of Medical Science.*

Sir—I am induced to communicate to you a few details of the Burmese practice of midwifery, in order that you may draw up and send me a set of plain rules and directions, such as the superior knowledge of anatomy and medicine professed by our physicians would point out, and I will have your rules translated into the Burmese language and circulated here. I know not a more benevolent act which I could perform.

About the seventh month of pregnancy a Burmese woman is advised to tie her *t' hamien* or petticoat more tightly and lower round the body, just above the *fœtus*, in order to force and keep it down as low as possible, and prevent its ascending; which, if it did, would afterwards, it is supposed, render delivery more tedious and difficult.

When the labor pains come on, the woman is attended by one or two *Woon-zwe* (midwives), and by three, four, five, or even six of her female relatives or friends, who shut all the doors and windows of the room, so as to render it as close and hot as possible. She is in a state of perfect nudity, and being urged to take violent exercise, runs round the room as long as she is able to do so, without or with the assistance of her friends, sometimes stopping and pressing her loins against the posts of the house, sometimes raising a heavy weight with both hands, and forcibly bringing it down, as if pounding paddy, and sometimes falling down and rolling on the floor. All this time also, she is uttering such loud and piercing cries and exclamations, as may be heard in the street and several doors off, vowing separation from her husband and wishing for death; which wish, the Burmese consider, is a proof of bad education and ignorance in any one expressing it. But the poor creature is quite distracted, and void of all sense of propriety, and sometimes grossly abuses her husband all the time. He is not admitted near her, and generally sits in the next room or in the street, laughing on hearing himself abused ; or if he possesses more feeling, he opens and lifts up the lid of every box in his house, as a preventive against any charm that may have been used by any evil disposed person, and prepares also, in a manner which I shall hereafter describe, some *charmed* or *holy* water, which he sends in to his spouse to drink.

The woman's body is smeared with oil, and her attendants, with many speeches of encouragement and comfort, such as, that she will not die, that all women bear children in the same way, &c., press down the child *violently* with their hands, urge the woman to strain, and sometimes put up a foot against her loins and press against her, holding her arms back.

At last the woman is quite exhausted, and falls on the floor. Some of the women still keep pressing the child down with their hands, trying to expel it forcibly ; and there are instances, I am credibly informed, in which the woman is placed on her back, and the midwife sits upon her, or stands up and presses against the child with one of her feet! Some of the other attendants, in the mean time, sit round the woman, and watch and notice, in so loud a voice as to be often heard in the street or adjoining houses, the appearance of the different parts of the child.

When the child is born, it is still kept near the mother until the after-birth comes away, to produce which the attendants again press the abdomen of the woman, pull the navel cord, and sometimes beat her loins with a hard pillow, and force a portion of her long hair down her throat, in order to create an inclination to vomit.

As soon as the after-birth appears, the navel string is cut, and the child taken charge of by one of the attendants, whilst the others, generally four of them, one to each arm and leg of the woman, take her up, bathe her in warm water, and place her as close to a large fire as possible, smearing her body with turmeric mixed with a little chunam, and making her swallow two and one-half ticals weight of salt, two of pounded turmeric, and a little chunam. A hot brick, and salt enveloped in a cloth, are also pressed against different parts of the woman's body in succession, and often a handful of warm salt is applied and *even introduced*.

This operation of exposure to fire, or rather roasting, as the Burmese women themselves call it (*mi-ken*) is one to which the woman is subjected for seven days, during which time, and oftener for a longer period, she is obliged to take the dose of salt and turmeric and chunam, in the proportions before mentioned, three times a day, at sunrise, noon and sunset, in order, it is said, to keep the inside of the body as hot as the outside ; and to drink warm water when thirsty, which of course she always is—and once or twice a day also, she is made to use a kind of vapour-bath, by sitting near the fire with a bamboo frame-work over her, covered with cloths steeped in hot water, or by sitting over a fire covered up, and sprinkling water upon the fire from time to time. She is often made also to sit on a heated brick covered with cloth. During the rest of the day and night, she lies on a plank or bamboo stage, raised five or six inches from the floor, and only a cubit wide, and placed as close to the fire as possible: she can only just turn her back or stomach to the heat, as she finds it too great on either side. The heat to which the woman is subjected, would be intolerable, but that she is every now and then smeared over with pounded turmeric and water. She is kept in a state of profuse perspiration, from which she is gradually relieved on the seventh day. A lady of rank, during these seven days, is known to have burnt as many as eleven hundred large billets of firewood, but the usual allowance is two or three hundred billets. The wood of the tamarind tree is also used by those who can afford it, as it is said to make the hottest fire. During the whole of this operation, no bandage is applied any where, and at the close of it, her skin is quite blackened, and peels off afterwards.

Some of the Burmese say, that a crab, half roasted, will not keep so well as when it is thoroughly done, and believe, that in the same way a newly delivered woman cannot expose herself too much to the fire. But from carelessness, or from the difficulty of shutting out draughts of air in a Burmese house, the poor woman often catches cold, and suffers from rheumatic affections of the limbs and other troublesome and lingering disorders; and whenever such cases of illness occur, the Burmese say, that they are owing entirely to the woman's not having been roasted enough! I have, however, heard many Burmese of respectability talk with horror of the customs of their country having subjected their women to such cruel sufferings, and attribute to this practice the cause of many women never having a second child. The principal midwife in this town, an active old Talain woman of 77 years of age, named *Mi-Ngyein* states, that she has followed her profession for more than fifty years, that she has delivered more than 10,000 women, that she is now often called to deliver the great-grand-daughter of one whom she had attended in early life, and that the average mortality, in her opinion, has been about ten per cent. This, however, must be much too high an estimate. Her usual charge is four or five rupees.

The diet of the woman during the first days after delivery, consists of boiled rice, with a kind of very hot broth, made by a mixture of the liquor of *Ngapee*, or fish sauce, a large quantity of pepper, some onions, and the root of a plant callee *k,hura*, remarkable for its heating properties. This soup she drinks by itself, as well as taking it with the rice, and it is so hot as to make her eyes run with water ; but it sensibly increases perspiration. During the roasting operation, also, a quantity of oil and salt is applied to the top of the head with the hair divided, and it is then held for some time as close to the fire as the woman can bear it. I am assured, however, that during this roasting operation, although the upper part of the body of the woman is always in a state of profuse perspiration, her feet and legs below the knees keep so cold, as to be unpleasant to the touch.

The mother is not supposed to have any milk for her infant, until after the third day ; and to produce the secretion, her breasts are rubbed and fomented with warm water, and the nipples pulled and scraped with the nails of her attendants. As soon as the child's navel string is separated, a quantity of pounded pepper is taken by one of the attendants in her forefinger, and rubbed all over the inside of the little creature's mouth, in order to make it throw up any phlegm or other matter which may be lodged in the throat or lungs. Sometimes a little boiled rice is masticated by one of the attendants, and forced down the child's throat ; but usually a little honey and water is given to it occasionally, which is its diet for the first three days, unless the child cries much, or the parents can afford it, when some woman who is nursing is called in to give the child the breast, until the mother can begin on the fourth day to nurse it herself.

Note.—This *charmed* or *holy water*, given to a woman in labor, is prepared by a person repeating seven times, over a cup of water, the following Pali words: *Yatau-hanbhaginee ariyaya Zatiya, Zatau nabhiza-nami thentseittsa panau Zeewita wauraupeta, tena thetstsena thotli te hautoo thatti gabat, tha*,—meaning, as I am informed by a Pali scholar, "O sister, from the moment of my having attained the state of an inspired priest of Boodh, I do not know a motive for depriving any sentient being of life, and as my words are true, mayest thou be at ease, as well as the being in thy womb." The Burmese have several *pareit* or prayers, used as a preventive of evil, but the above is called *Engooli Mala pareit*, and was dictated by *Gaudama* under the following circumstances.

A Bramin named *Aheintha-ka* of *Thawotthi*, (*Spavasti* in Oude,) applied for instruction in learning to a celebrated teacher in the city of *Tekkatho*, named *Deitha Pamoukha*. Being a man of bad and cruel character, the teacher was unwilling to instruct him, and proposed to receive him as a pupil, upon condition only that he should present the teacher with one thousand human forefingers, thus setting the Bramin, as Samson of old had been set, a task, the execution of which, it is hoped, would cost him his life. The Bramin, however, proceeded to attack, men, women and children, and killing them, cut off their forefingers, and hung them in a string round his neck, whence he was afterwards known always by the name of *Engolee-Mala*, necklace of fingers. He had collected 999 forefingers, and was in the act of chasing his own mother to kill her, and complete his task, when *Gaudama* interposed between them, and converting the Bramin into a Buddhist disciple, made him a priest, and lodged him in the same monastery with himself, near the city of *Thawotthi*. *Engolee-Mala*, however, had become the dread of the whole country around, and the cry that he was coming, or sound of his very name, terrified women and children, and made pregnant women miscarry. For some time after he had been converted by Gaudama, whenever he appeared in the streets of Thawotthi, as he did every morning to receive charitable offerings, according to the custom of Buddhist priests, the women and children fled before him, and the men chased him with stones. Having been much bruised one day, he applied to Gaudama for protection, and he delivered to him the foregoing Pali words, desiring him to repeat them whenever he saw any woman, and assuring him that they would save him from all further molestation. The words operated as a charm, and they are now always used as a preventive of evil. They are considered of such wonderful efficacy, that the water with which any spot, on which a person may have been sitting or standing whilst reciting the words, is washed, can charm away evil and danger.—*Ib.* A. F.

23. *Experiments upon the means of producing an artificial Cataract, and thus facilitating the study of Opthalmology.*—Several persons have undertaken to produce an artificial cataract, by submitting the eye to the action of some reagent, or of introducing one by means of a convenient instrument, so as to coagulate the crystalline lens. Troja found that the lens became opaque, when he surrounded the eye with unpurified marine salt, slightly moistened; but the cornea often became opaque at the same time. He used nitric acid diluted with ten times its weight of water, which also obscured the cornea, but it was only superficial, and disappeared by raising a pellicle, beneath which the cornea appeared perfectly transparent. M. Serre, the author of this paper, has turned much attention to this subject, and has made many experiments on it. He obtained thirty rabbits, whose eyes are most analogous to the human eye. He found that the most simple and easy means consisted in wounding the lens with a fine needle, penetrating through the cornea. After twenty-five days or a month he obtained, in a majority of the animals, a very uniform cataract, without lesion of the iris or retina: in some the cataract was partial, and extended but a little around the wounded point; in some the pupil contracted and became immoveable, by the adhering of the iris with the opaque crystal, and in three cases, there was total obliteration of the pupil. A butcher brought to M. Serre the eye of a sheep. After examining the iris, he discovered an opaque crystaline lens. He placed it about a foot from the fire, on a piece of paper, to keep for further examination. At the end of a quarter of an hour, he found it had resumed its transparency. He then tried several experiments with it, connected with a thermometer, and he found when its temperature sunk to 17° cent. its opacity returned quite thick, at 10° it was a very thick white, and presented a beautiful cataract. On exposing it before a fire, where the mercury rose to 20°, the opacity began to disappear, and at 25° it was transparent.—*U. S. Medical and Surgical Journal.*

24. *New process for the operation Artificial Pupil.*—M. Laugier, of the Hospital Necker, had occasion to make an artificial pupil in a man of sixty years. The intention of the surgeon was to seize the central part of the iris with a very slender crotchet, and excise it in its place, if he could not draw it between the edges of the cornea. But the aqueous humor was entirely driven out by a sudden movement of the eye, before the knife of Richter could complete the incision. M. Laugier conceived the happy thought of completing the operation in the following manner: he introduced the depressing needle, with the point directed towards the centre of the iris, with which he acted upon the centre as a lever, and produced without hœmorrhage and almost without effort, a perforation, by tearing. The patient completely recovered the sight of his eye. The artificial pupil is irregular and quadrangular, so that it is probable the needle has not only broken the central adhesions of the iris, but has also torn a point of the circumference of this membrane.—*Medical and Surgical Jour.*

25. *A case of Introsusception, in which an operation was successfully resorted to by* John R. Wilson, M. D. Communicated by Mr. W. W. Thompson, Student of Medicine.—The subject of the operation was a negro man, aged about twenty years, the property of Mr. Charles Dement. The patient had labored for seventeen days under bilious colic, and stercoraceous vomiting, and the other more alarming symptoms of this disease, had appeared. All the active purgatives were administered in vain, and, on the evening before the operation was resolved upon, as a dernier resort, some ounces of crude mercury were given. The constipation remaining, with the other formidable appearances, it was plain that nothing but the knife could save the patient.

The operation was performed in the following manner. An incision was made along the linea alba, commencing above the umbilicus and extending two or three inches below it, being in all about five inches in extent. The bowels being protruded through the wound, that portion involved in the stricture came into view. It was found to be in the *ileum.* The bowel was grasped above and below the point of obstruction, and after several efforts of considerable force, the adhesion gave away. The exertion necessary to break up the attachments, it was feared, might lacerate the intestine; but no such accident followed. The bowel strangulated was of a dark lived appearance, evidently approaching to gangrene, and of double its ordinary size. The vessels of the omentum were also deeply engorged with black blood, apparently stagnant. The parts seemed to be on the verge of mortification. After returning the intestines into the abdomen, having carefully excluded the atmosphere during the operation by a warm, moist cloth spread over the viscera, the wound was made secure by a few stitches with the needle, and adhesive strips. The patient was put to bed, and in a very short time voided the mercury which he took the evening before. His recovery was rapid and entire.

The success of this case, in which the operation was so long deferred, and at last performed under such unfavorable circumstances, warrants the propriety of resorting to it in the disease, and proves that relief may occasionally be afforded by this means, when all others have failed.—*Transylvania Journal of Medicine.*

26. *On Delirium Tremens.* By J. Young, M. D.—"To those who are about to enter on the treatment of disease, and have never met with a case of mania a potu, or delirium tremens, I would say, when you are called to a case of it, bear in mind the axiom, that an old drunkard will not bear direct depletion to the same extent as other persons, under what may appear to be very similar circumstances. If you determine on the use of the lancet, keep your finger on the pulse, and the moment you find its force and fulness diminishing, and its frequency increasing, close the orifice; having done this, under the same view of the state of the system, prescribe exactly as your judgment shall teach you to be proper for the existing state of affairs; but believe it not, when you are told to commence, in all cases, at once with mammoth doses of opium, and that it never did, never will, and never can do harm in this disease. In the hands of Dr. Coates, whose extensive experience, and refined judgment, enables him to discriminate at a glance, exactly the case which consists in pure nervous mobility, or cerebral irritability, opium is the sovereign remedy. But you will find few cases indeed, in actual practice, of this purely uncomplicated character; and if your acumen enables you to recognize the local affection, and your remedies *can subdue* it, you will cure your patient; but if you lose him, and post mortem examintion shall disclose to you the fact, that you treated him for the disease he in reality labored under, how satisfactory will be the reflection that you did all that human art could do for him, when compared with the mortifying feelings that must result from a different autopsic revelation."—*United States Medical and Surgical Journal.*

27. Dr. Graves *on the Chloride of Sodium in Fever.*—In relation to the time for its exhibition, and the cases to which it is adapted, the following remarks may be made. When the early stage of fever is past, when all general and local indications have been fulfilled, when there is no complication with local disease, when the patient lies sunk and prostrated, when restlessness, low delirium, and more or less derangement of sensibility is present, when the body is covered with maculæ, and when the secretions from the skin and mucous membranes give evidence of a depraved state of the fluids, it is then that the chloride of sodium may be prescribed with the most decided advantage. The mode in which I prescribe it is in doses of from fifteen to twenty drops every fourth hour, in an ounce of water or camphor mixture. How it acts I will not pretend to explain; it is sufficient to say, that there is no remedy from which, in such cases, such unequivocal benefit is derived. It operates energetically, though not very rapidly, in controlling many of those symptoms which create most alarm. It seems to counteract the tendency to tympanitics, to correct the fetor of the excretions, to prevent collapse, to promote a return to a healthy state of the functions of the skin, bowels, and kidneys; in fact, it appears admirably calculated to meet most of the bad effects of low putrid fever. To those who have witnessed its efficacy, it is unnecessary for me to say any thing. Of course it will fail, like all other remedies, when the disease has reached a certain point of intensity in individual cases. There is scarcely any acute disease, to which the human body is liable, which may not in some particular persons assume an intensity capable of baffling all the effort of medical skill. This, however, is no argument against the employment of a remedy of extensive utility and unquestionable value.

Although it is not my intention to give an account of what has been done in France with respect to the exhibition of this remedy, yet I may mention, that it has been extensively tried in fever by Chomel, and as I have learned with great success. This excellent physician is still, I believe, engaged in making further clinical experiments on the subject. In the *Gazette Medicale de Paris*, published on the 28th of last February, we have an account of Dr. Dor, of Marseilles, of several cases of typhus, in which the chloride of sodium was found beneficial in 1833. He attributes a more rapid amendment to the use of this remedy than I have even seen follow from its exhibition, and he also asserts, that if not given with great caution, it produces a very tedious convalescene. In the latter remark, especially, I cannot concur: for all who witnessed this mode of treatment here, were struck with the security and quickness of recovery which ensued in those cases where it had been employed. Perhaps the precaution we adopted of always diminishing, as soon as possible, the strength and frequency of doses, rendered the results in our hands more satisfactory than those obtained by Dr. Dor."—*Medico-Chirurgical Review.*

28. *Typhus in the Young.*—Four cases of typhus in children, occurring in the Hospital des Enfans Malades. Paris, are related in the London Lancet, appended to which are the following remarks.

The above cases cannot be arranged under any other head than typhus fever; although, so far as we know, this latter affection has never yet been described by systematic writers as attacking children at a very early period of life. However, the more we see of the diseases of children, the more we are convinced of the truth of the proposition laid down by M. Guersent, viz: that children are subject to all the diseases of adults, and, in addition, to certain maladies which are peculiar to their tender age. Thus, in the course of November last, we had occasion to observe the case of a boy, eight years of age, affected with an urinary fistula at the root of the scrotum, not the result of an accidental lesion, but produced in a gradual manner, like the fistula caused by stricture in the adult. Another case, not less rare, presented itself on the 18th of December, 1835. A boy, fourteen years of age, was brought to the hospital, in a state of extreme weakness; pulse 100; violent pain of the abdomen; twenty to twenty-four stools in the day. The patient died in a few hours after his admission, and, on examining the body, the abdominal aorta was found to be the seat of three large false aneurisms; the superior of which, placed nearly on a level with the kidney, had given way, to the extent of a couple of inches. About the same time, a case of cancer of the abdomen was observed in the "service" of M. Jadelot, in a child six years of age.

The four cases we have detailed are sufficient to indicate the general characters of typhus in the child; the symptoms, indeed, seem to differ very little from those which mark the disease in the adult; there is the same prostration of strength, the same derangement of the intelligence without any sign of cerebral inflammation—in a word, the same tendency to adynamic and ataxic symptoms; however, the affection is, generally speaking, a much milder one in the child than in the adult; the mortality is much less for the former than for the latter, but the march of the disease is precisely the same, and after death we find the same lesions in the intestinal canal.

The treatment adopted at the *Hospital des Enfans Malades* is, in general, extremely simple, and differs widely from that which is pursued in England. M. Guersent commences by applying a few leeches to the abdomen, (if the belly be at all tender,) and pursues what may be strictly called the "expectant method," enjoining diet, cooling drinks, and a sinapism now and then to the legs. If, however, ataxic symptoms manifest themselves, he orders a trepid bath, with cold affusion on the head, and applies blisters to the thighs instead of sinapisms. The adynamic symptoms are rarely treated until they are very far advanced. A lavement, with quinine, is then thrown up the rectum, and a few spoonfuls of Bordeaux or Malaga wine are given every now and then.

M. Baudelocque is the only physician who employs the purgative method, so much in vogue in England since the publication of the work of Hamilton. The medicament he administers in preference to others is Seidlitz water. The advantage of purging, however, has appeared very doubtful, especially in the last few months, during which a tendency to dysentery has prevailed amongst the patients. In several cases the administration of a few spoonfuls of Seidlitz water, determined severe purging and dysenteric symptoms, which eventually carried off the patient. It is hardly necessary for us to make any farther remarks on this subject; we shall therefore merely observe, in conclusion that, on examining the bodies of children which have been cut off by this disease, we have never yet discovered the least trace of inflammation in the cerebro-spinal system. The stupor, the delirium, and the convulsive movements, are merely sympathetic phenomena, and certainly are not connected with an organic change of the brain; yet how often do we see the whole attention of the physician directed to these symptoms.—*Lond. Lancet —Boston Med. and Sur. Jour.*

